# The conserved ubiquitin-like protein Hub1 plays a critical role in splicing in human cells

**DOI:** 10.1093/jmcb/mju026

**Published:** 2014-05-28

**Authors:** Tim Ammon, Shravan Kumar Mishra, Kaja Kowalska, Grzegorz M. Popowicz, Tad A. Holak, Stefan Jentsch

**Affiliations:** 1 Department of Molecular Cell Biology, Max Planck Institute of Biochemistry, Am Klopferspitz 18, 82152 Martinsried, Germany; 2 NMR Spectroscopy, Max Planck Institute of Biochemistry, Am Klopferspitz 18, 82152 Martinsried, Germany; 3 Present address: Department of Biological Sciences, Indian Institute of Science Education and Research (IISER) Mohali, Knowledge City, Sector 81, SAS Nagar, 140306 Punjab, India; 4 Present address: Institute of Structural Biology, Helmholtz Zentrum München, Ingolstädter Landstrasse 1, 85764 Neuherberg, Germany; 5 Present address: Faculty of Chemistry, Jagiellonian University, Ingardena 3, 30-060 Cracow, Poland

**Keywords:** apoptosis, Hub1, splicing, spliceosome, ubiquitin-like proteins

## Abstract

Different from canonical ubiquitin-like proteins, Hub1 does not form covalent conjugates with substrates but binds proteins non-covalently. In *Saccharomyces cerevisiae*, Hub1 associates with spliceosomes and mediates alternative splicing of *SRC1*, without affecting pre-mRNA splicing generally. Human Hub1 is highly similar to its yeast homolog, but its cellular function remains largely unexplored. Here, we show that human Hub1 binds to the spliceosomal protein Snu66 as in yeast; however, unlike its *S. cerevisiae* homolog, human Hub1 is essential for viability. Prolonged *in vivo* depletion of human Hub1 leads to various cellular defects, including splicing speckle abnormalities, partial nuclear retention of mRNAs, mitotic catastrophe, and consequently cell death by apoptosis. Early consequences of Hub1 depletion are severe splicing defects, however, only for specific splice sites leading to exon skipping and intron retention. Thus, the ubiquitin-like protein Hub1 is not a canonical spliceosomal factor needed generally for splicing, but rather a modulator of spliceosome performance and facilitator of alternative splicing.

## Introduction

Ubiquitin family proteins (ubiquitin, SUMO, Rub1/Nedd8, etc.) are central regulators of cellular functions (Hochstrasser, 2000). Canonical members of this protein family are enzymatically and reversibly conjugated to other proteins, thereby functioning as covalent protein ‘modifiers’. Although structurally very similar to ubiquitin (McNally et al., 2003; Ramelot et al., 2003), the highly conserved protein Hub1 does not function as a covalent modifier but binds proteins only non-covalently (Luders et al., 2003; Yashiroda and Tanaka, 2004; Mishra et al., 2011). In *Saccharomyces cerevisiae*, Hub1 binds tightly to the spliceosomal protein Snu66, a protein of the U4/U6.U5 small nuclear ribonucleic particle (tri-snRNP) (Wilkinson et al., 2004; Mishra et al., 2011). However, contesting earlier reports (Dittmar et al., 2002; Wilkinson et al., 2004), we have shown previously that yeast Hub1 is not required to localize Snu66 to the nucleus, but that Hub1 affects splicing directly through non-covalent interactions (Mishra et al., 2011). Snu66 of *S. cerevisiae* possesses near its amino (N)-terminus two tandem-arranged Hub1 interaction domains, termed HIND, enabling the splicing factor to bind up to two Hub1 molecules (Mishra et al., 2011). In *S. cerevisiae*, Hub1 is not essential for viability and is apparently also not generally required for splicing as judged by splicing-sensitive microarray assays (Mishra et al., 2011). Intriguingly, *hub1Δ* cells fail to promote alternative splicing of *SRC1*, which is one of the rare cases of *S. cerevisiae* genes for which alternative splicing has been reported. Abolishing Hub1–Snu66 interaction by mutation affects *SRC1* alternative splicing as well (Mishra et al., 2011), suggesting that binding of Hub1 to Snu66 is critical for Hub1’s function in *S. cerevisiae*. Since also *SRC1* is not essential for viability, it seems possible that the function of Hub1 of *S. cerevisiae* is restricted to *SRC1*. In contrast, in *Schizosaccharomyces pombe*, in which splicing is much more prevalent than in *S. cerevisiae*, Hub1 affects splicing of several pre-mRNAs and is essential for viability (Yashiroda and Tanaka, 2004; Mishra et al., 2011).

Contrasting these detailed findings, much less is known about Hub1 from higher eukaryotes. Human Hub1 (also known as UBL5 or beacon) appears to be exported from the nucleus upon hypo-osmic shock (Hatanaka et al., 2006), and known to bind certain protein kinases (Kantham et al., 2003). Hub1 has been detected in human spliceosomes by mass spectrometry (Deckert et al., 2006) and reported to be implicated in pre-mRNA splicing (Švéda et al., 2013; Laetsch et al., 2014), but a detailed characterization of the cellular function of mammalian Hub1 was lacking. In the nematode *Caenorhabditis elegans*, Hub1 was identified in a genetic screen for genes implicated in the unfolded protein response in mitochondria (UPR^mt^) (Benedetti et al., 2006). Moreover, co-immunoprecipitation experiments from cell extracts suggested that *C. elegans* and mammalian Hub1 associates with the DVE-1 transcription factor responsible for the UPRmt pathway (Haynes et al., 2007). However, whether Hub1 binds the transcription factor directly and controls transcription has not been tested. Similarly, the mammalian homolog of Snu66, termed hSnu66 or SART1, has been suggested to modulate transcription as well (Gupta et al., 2000), but *in vitro* splicing assays with human nuclear extracts have shown that hSnu66 is crucial for splicing and present in spliceosomes as in yeast (Makarova et al., 2001; Liu et al., 2006; Bessonov et al., 2008).

Here we address the cellular role of human Hub1 biochemically as well as functionally by siRNA-mediated depletion. Our study revealed a strong conservation of Hub1 and its binding to Snu66 at the molecular level. However, we found that Hub1 is much more important for human cells than for *S. cerevisiae*, and also describe that Hub1 depletion causes splicing speckle abnormalities and mitotic defects culminating in caspase-mediated apoptosis. Importantly, Hub1 does not seem to influence splicing of all splice substrates equally, but to facilitate only certain splicing events of particular introns/exons of pre-mRNAs. These findings thus lead to the model that the ubiquitin-like protein Hub1 plays a conserved role in the spliceosome as modulator of splicing activity.

## Results

### Human Hub1−Snu66 complex

**Figure 1:**
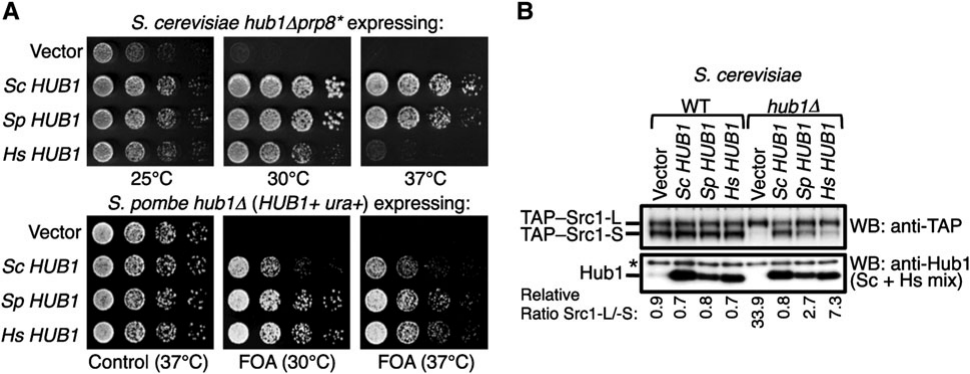
Conserved and divergent properties of Hub1. (**A**) Genetic complementation assays. Rescue of synthetic sickness of hub1Δprp8* in *S. cerevisiae* (top panel) and lethality of *hub1Δ* in *S. pombe* (bottom panel), by expression of Hub1-encoding genes (or cDNAs) from *S. cerevisiae* (*Sc*), *S. pombe* (*Sp*), and *H. sapiens* (*Hs*). For complementation in *S. pombe*, a *URA4*-bearing plasmid expressing WT SpHUB1 was shuffled-out from the *hub1Δ* strain by counter-selection with FOA. Growth assays with 5-fold serial dilutions on control or FOA-containing plates at indicated temperature are shown. (**B**) Complementation of altered alternative splicing of *S. cerevisiae SRC1* in hub1Δprp8* cells by HUB1 orthologs at 30°C (like in **A**). Protein expression levels of TAP-tagged *Src1*-L and *Src1*-S isoforms as well as Hub1 were monitored by immunoblotting using anti-TAP and anti-Hub1 antibodies, as described previously (Mishra et al., 2011). The quantification of the relative ratio between *Src1-L* and *Src1-S* isoforms is given below.

To address whether human Hub1 is involved in splicing, we first asked whether expression of human Hub1 could complement the phenotypes of the Hub1 deletion mutants of *S. cerevisiae* and *S. pombe*. Since *S. cerevisiae* strains with a deletion of the Hub1-encoding gene (*hub1Δ*) are viable and cells exhibit no discernable growth defects, we rather assayed in a genetic background (prp8*, partially defective in the spliceosomal protein Prp8) in which Hub1 becomes essential for viability (Mishra et al., 2011). Notably, although both *S. pombe* and human Hub1-encoding genes could rescue the synthetic lethality of the hub1Δ prp8* double mutant, human Hub1 was unable to do so at higher temperatures (Figure 1A; top panel; for protein levels see Supplementary Figure S1A). Moreover, the defect in alternative *SRC1* splicing of the *S. cerevisiae hub1Δ* mutant (Mishra et al., 2011) was considerably rescued by *S. pombe* Hub1 but only weakly by human Hub1 (Figure 1B). Conversely, when we assayed for complementation of the *S. pombe hub1Δ* mutant, we found that expression of human Hub1 rescued the lethality of this mutant like *S. pombe* Hub1, whereas expression of the *S. cerevisiae* gene provided viability, yet the strain exhibited a mild growth phenotype (Figure 1A; bottom panel; for protein levels see Supplementary Figure S1B). Thus, echoing the sequence divergence of the various Hub1 proteins, human and *S. pombe* Hub1 are functionally similar, whereas *S. cerevisiae* Hub1 is divergent to some degree.

**Figure 2:**
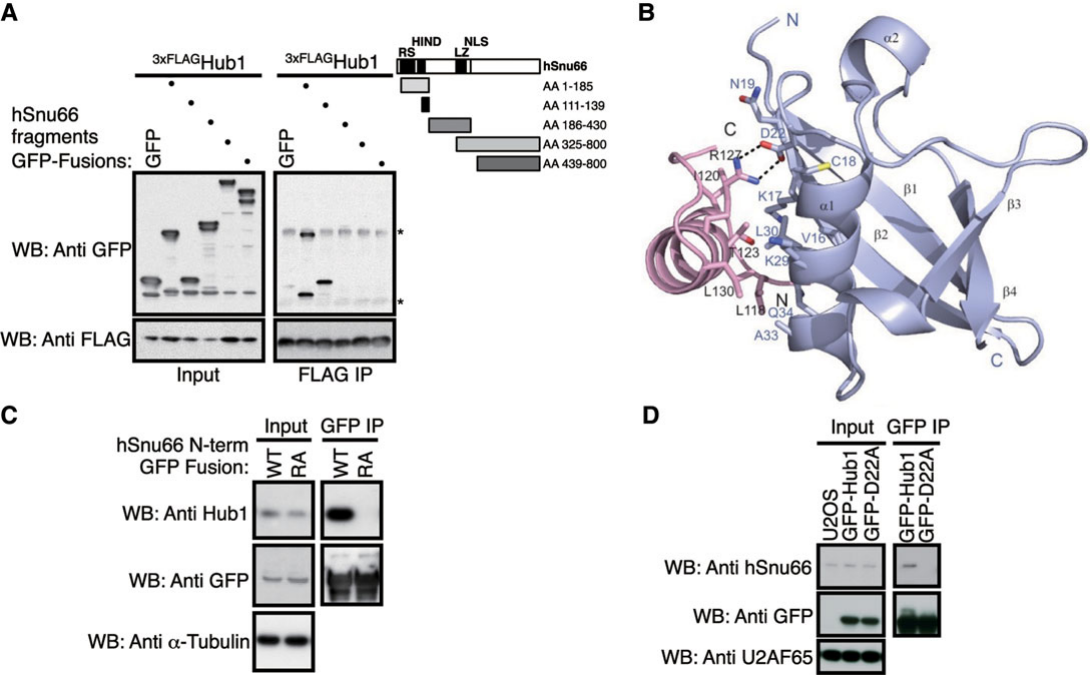
Molecular mode of interaction between human Hub1 and HIND. (**A**) Mapping of the Hub1 interaction domain in hSnu66 using FLAG-immunoprecipitation of 3xFLAG-Hub1 after co-expression of GFP-tagged hSnu66 truncations or free GFP in human cells. Immunoprecipitates were immunoblotted with anti-FLAG and anti-GFP antibodies (Asterisks indicate light and heavy chains). (**B**) Crystal structure of human Hub1 (blue) in complex with HIND peptide (pink) of hSnu66 shown as a ribbon plot with a resolution of 2.0 Å. The interaction between Hub1 and the α-helical HIND peptide is mediated through a salt bridge formed by D22 of Hub1 and R127 of HIND, strengthened by hydrophobic contacts involving aliphatic fragments of residues of hSnu66's HIND (L118, I120, T123, L126, R127 (Cβ and Cγ), L130, L132, L135) and the Hub1 interface (M1, V16, L17 (Cβ, Cγ, Cδ), C18, N19 (Cβ, Cγ), L29 (Cβ, Cγ, Cδ), L30, A33). (**C**) GFP-directed immunoprecipitation of GFP fused to the HIND containing N-terminal domain (aa 1–139) of wild-type hSnu66 (WT) or Hub1-binding-deficient HIND mutant (R127A). Immunoblot detection using antibodies directed against GFP, human Hub1, or α-tubulin (control). (**D**) Co-immunoprecipitation of Hub1 with hSnu66 depends on the HIND interaction interface. GFP immunoprecipitation from U2OS cells stably expressing GFP-tagged Hub1 WT or hSnu66 binding mutant Hub1 D22A. Immunoblots were probed with anti-GFP and anti-hSnu66 antibodies with anti-U2AF65 serving as a loading control.

Hub1 of yeast and mammalian cells associates with the spliceosome through interaction with the tri-snRNP protein Snu66 (Mishra et al., 2011). Unlike *S. cerevisiae* Snu66, which possesses two tandem-arranged HIND elements in its N-terminal domain, *S. pombe* and human Snu66 proteins harbor only one element (Mishra et al., 2011). In contrast to its *S. cerevisiae* counterpart, human Snu66 (referred to hSnu66 in the following) harbors an arginine/serine rich (RS) domain (aa 41–108) directly N-terminally of its HIND motif (Makarova et al., 2001). Because RS domains can mediate protein–protein interactions as well (Wu and Maniatis, 1993; Wang et al., 1995), we mapped the Hub1-binding site using hSnu66 truncations and found that the single HIND motif of hSnu66 is sufficient and necessary for Hub1 binding (Figure 2A and Supplementary Figure S2A).

To characterize the Hub1–HIND interaction further, we obtained structural information of human Hub1 in complex with a peptide corresponding to the single human HIND sequence (Figure 2B and Supplementary Figure S2B). The solved crystal structure (PDB code 4PYU) with a resolution of 2.0 Å highlights the typical ubiquitin β-grasp fold of human Hub1, with the typical ββαβαβ secondary structure pattern, as described previously (McNally et al., 2003; Ramelot et al., 2003). The interaction between Hub1 and the α-helical HIND peptide is mediated through a salt bridge formed by D22 of Hub1 and R127 of HIND, strengthened by hydrophobic contacts involving aliphatic fragments of residues of hSnu66’s HIND and the Hub1 interface (Figure 2B). Although Hub1 possesses an ubiquitin fold, it uses the opposite protein surface for Snu66 binding compared to ubiquitin for binding to ubiquitin receptors like Rad23 (Mishra et al., 2011). The X-ray analysis revealed that human and *S. cerevisiae* Hub1-HIND complexes have overall very similar, superimposable structures with a root mean squared deviation (RMSD) of 0.716 Å. The two structures only show small differences, particularly in protein loops extending opposite of the Hub1–HIND interaction surface (Supplementary Figure S2C).

Testing the relevance of the salt bridge between D22 of Hub1 and R127 of the HIND element for this interaction, we found that a recombinant fusion protein of GFP with the N-terminal domain of hSnu66 (aa 1–139), but not a similar fragment bearing an alanine replacement of a the R127 residue (R127A) in its HIND domain, bound endogenous Hub1 of human cell extracts in GFP-immunoprecipitation assays (Figure 2C). Conversely, only immunoprecipitation of stably expressed GFP-fusions of wild-type (WT) Hub1 but not of a Hub1 variant harboring an alanine replacement of the D22 residue (D22A) co-isolated endogenous hSnu66 from human cell lysates (Figure 2D). From these findings we infer that binding of human Hub1 to Snu66 does indeed depended on the integrity of this salt bridge, showing that physical properties of Hub1 are highly conserved at the molecular level.

### Hub1 is essential for viability of human cell lines

**Figure 3:**
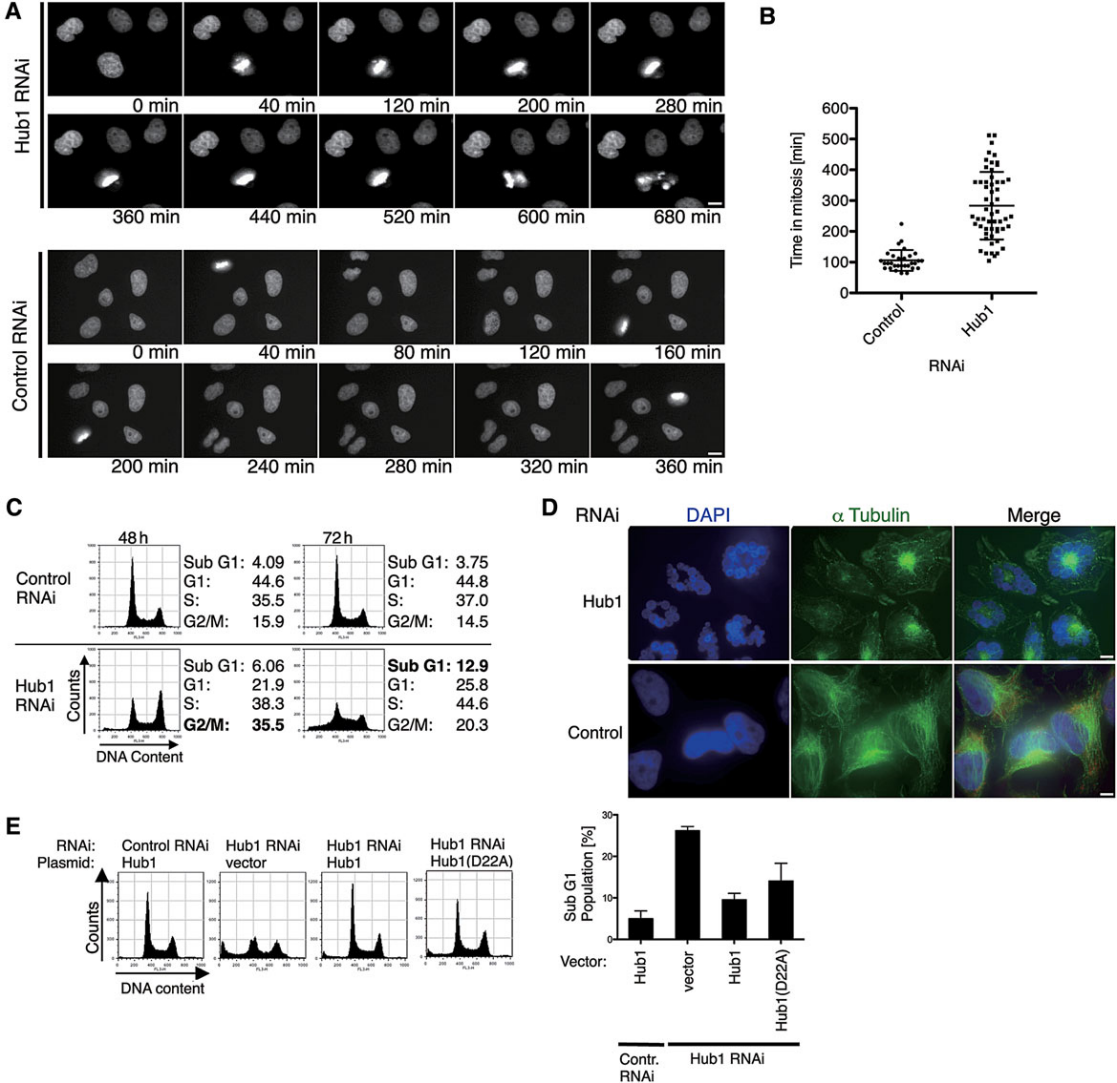
Human Hub1 is essential for viability. (**A**) Live cell microscopy of H2B-GFP HeLa cells treated with RNAi against Hub1 or non-targeting control. The images represent stills of time-lapse video microscopy at representative time points 60 h post transfection. Scale bar, 10 µm. (**B**) Quantification of cell cycle delay after Hub1 knockdown by measuring time in mitosis from nuclear envelope breakdown until completion of mitosis by live cell microscopy of H2B-GFP HeLa cells. Data represent mean and standard deviation (SD) for control RNAi (*n* = 30) and Hub1 RNAi (*n* = 56). (**C**) Flow cytometry analysis of cell cycle distribution and induction of apoptosis after Hub1 depletion 48 or 72 h post transfection. Quantifications for the sub-G1 fractions are shown next to the flow cytometry profiles. (**D**) Representative images of HeLa cells 72 h after RNAi transfection exhibiting loss of nuclear integrity and structural abnormalities. In contrast to wild type or control RNAi-treated cells where nuclei were integer and regular in shape with a typical outspread α-tubulin network, Hub1 RNAi-treated cells exhibited deformed and disintegrated nuclei, segmented into multiple micronuclei that were radially arranged around central dense α-tubulin material. Immunofluorescence staining with anti-α-tubulin antibodies (green) and DAPI visualizes structural abnormalities and nuclear rearrangements. Scale bar, 10 µm. (**E**) Complementation of Hub1 RNAi by expression of siRNA-resistant Hub1 encoding WT or Hub1 D22A mutant. Analysis of cell cycle distribution and induction of apoptosis by flow cytometry with quantification of apoptotic sub-G1 fraction in Hub1 complementation assays (right panel, data represent mean and SD of three independent experiments). Due to highest knockdown efficiency Hub1 depletion and complementation experiments were performed using siRNA oligo iHub1_1.

We next addressed the cellular importance of human Hub1 by *in vivo* depletion of Hub1 from human cells using RNAi. Transfection of Hub1 siRNA led to an efficient depletion of the Hub1 protein in cell cultures (Supplementary Figure S3A). Live cell imaging revealed that ∼48 h after siRNA treatment, cells start to exhibit strong cell cycle progression delays, accompanied by defects in mitotic cell division, for instance in metaphase plate formation and chromosome segregation (Figure 3A and B). Flow cytometry analysis showed that Hub1 siRNA treatment initially (after 48 h) caused defects in G2/M cell cycle progression, and culminated later (72 h) in a rise of sub-G1 fractions, indicative of cells undergoing apoptosis (Figure 3C). We also induced cell cycle arrest in S-phase in Hub1 siRNA-treated cells using a double thymidine block, and monitored synchronous cell cycle progression after release from the block by a washing step. Again we found that Hub1 siRNA-treated cells, but not cells treated with a control siRNA, exhibited G2/M cell cycle progression defects ∼9 h after S-phase release (Supplementary Figure S3B). Prolonged Hub1 siRNA treatment led to aberrant mitosis with characteristics of mitotic catastrophe (mitosis-linked cell death) as indicated by live cell imaging as well as α-tubulin staining (Figure 3A and D). The observed defects included chromosome mis-segregation and loss of nuclear integrity, which gave rise to segmented nuclei and later apoptosis.

Indeed, apoptosis was caspase-dependent and affected the majority of Hub1 siRNA-treated cells as indicated by the efficiency of caspase-7 cleavage and annexin V/PI stainings (Supplementary Figure S3C and D). Transfection of an siRNA-resistant Hub1 cDNA restored WT phenotypes to Hub1 siRNA-treated cells (Figure 3E and Supplementary Figure S3E), verifying that the observed phenotypes were caused by Hub1 depletion. Notably, a siRNA-resistant cDNA expressing a Hub1 variant deficient in Snu66 interaction (D22A) rescued viability only partially, indicating that binding of Hub1 specifically to the spliceosomal protein hSnu66 might contribute, but may not be as critical in humans as in *S. cerevisiae*.

### Depletion of human Hub1 causes splicing speckle abnormalities

**Figure 4:**
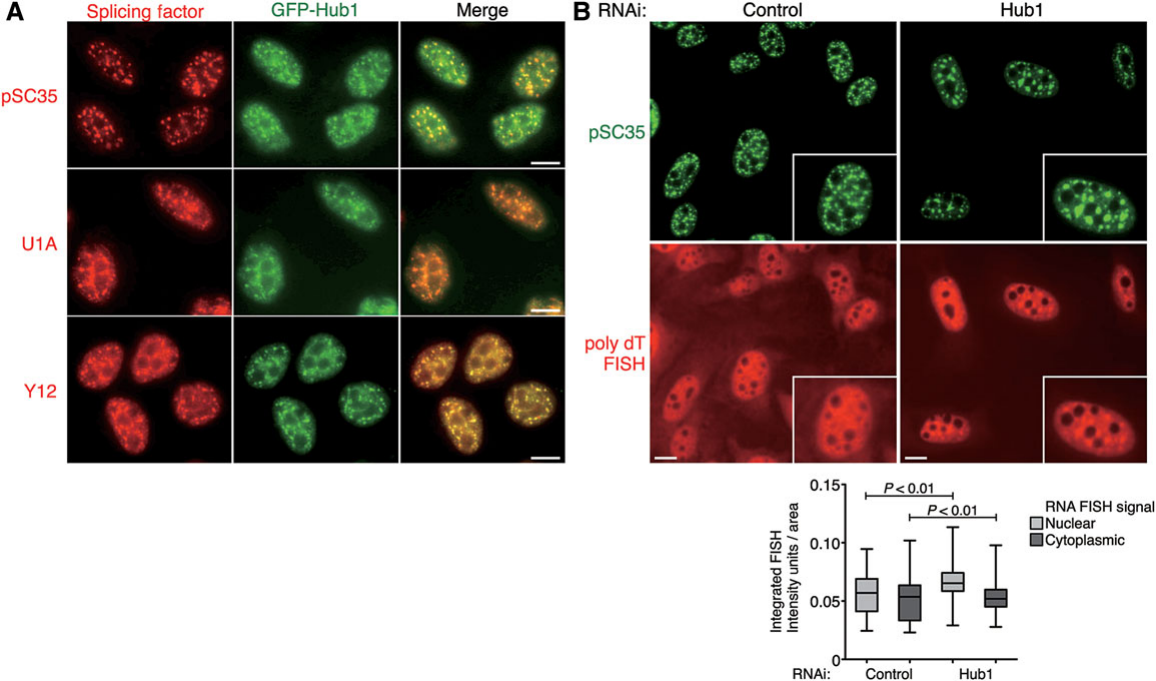
Depletion of Hub1 causes nuclear speckle abnormalities. (**A**) Co-localization studies in U2OS cells stably expressing GFP-Hub1 (green). Cells were pre-extracted, fixed, and immunostained for splicing proteins (red) using antibodies against nuclear speckle marker phospho-SC35, U1 snRNP (anti-U1A antibody), and snRNP associated Sm proteins (anti-Y12), respectively. Scale bar, 10 µm. (**B**) Visualization of poly-adenylated mRNA by FISH with fluorescently labeled poly-(dT)-TRITC probes co-stained for nuclear speckles with anti-SC35 antibodies in U2OS cells treated with Hub1 or control RNAi. Scale bar, 10 µm. The nuclear accumulation of poly-adenylated mRNA upon Hub1 knockdown is quantified by measuring the integrated FISH signal as arbitrary intensity units per area in nuclear and cytoplasmic compartments. The box-and-whisker plots represent the quantification of two independent experiments with significant differences between Hub1 and control RNAi treated cells (*P* as the probability of a two-tailed paired *t*-test, *n*> 140 for control cells and *n* > 239 for Hub1 knockdown cells).

The physical interaction of Hub1 with spliceosomal components like hSnu66 and SR-protein Cdc2/cdc28-like kinases (Clk) (Kantham et al., 2003) emphasizes the link between Hub1 and the pre-mRNA splicing machinery. Localization studies using immunofluorescence microscopy showed that Hub1 resides in so-called splicing speckles, i.e. splicing factor-associated nuclear assemblies that appear microscopically as irregular punctate nuclear structures (Lamond and Spector, 2003). In these splicing speckles, human Hub1 co-localizes with characteristic nuclear speckle markers, like the serine/arginine-rich (SR) protein SC35 (alias SRSF2; Huang and Spector, 1992), the U1 snRNP (identified with anti-U1A antibody; Sleeman et al., 1998), and snRNP-specific Sm proteins (identified with Y12 antibody; Lerner et al., 1981) (Figure 4A). Our previous work showed that hSnu66 is able to recruit Hub1 to nuclear speckles via its HIND element upon transient Hub1 and hSnu66 co-overexpression (Mishra et al., 2011). Yet, when Hub1 was stably expressed, nuclear speckle localization of Hub1 (GFP-Hub1) was largely normal even when Hub1 was deficient in HIND interaction (D22A mutant variant; Supplementary Figure S4A). Thus, human Hub1 is able to associate with splicing speckles also independently of Snu66. This finding supports the above observation (Figure 3E) that binding of Hub1 to Snu66 is not essential for Hub1 function, and suggests that other surfaces of Hub1 may contribute to splicing factor association.

Splicing speckles are typically highly dynamic as some of their protein and RNA content cycle continuously between speckles, sites of transcription and other nuclear locations (Misteli et al., 1997; Politz et al., 2006; Spector and Lamond, 2011). The splicing protein SC35, for example, also associates with sites of active transcription where it promotes transcriptional elongation via binding to the C-terminal domain of RNA polymerase II (Lin et al., 2008). Moreover, splicing and mRNA export are often coupled (Jiménez-García and Spector, 1993) and hence partly spliced mRNAs accumulate in the nucleus upon splicing inhibition (Kaida et al., 2007). Because of Hub1's association with components of the splicing machinery in nuclear speckles and the observed strong phenotypes associated with Hub1 depletion in human cells at later stages of knockdown, we asked whether Hub1 influences the structure of splicing speckles and the nuclear shuttling of mRNA. Indeed, when we transfected Hub1 siRNA into cells, the SC35, 3mG cap (2,2,7-trimethylguanosine cap of non-U6 snRNPs) and U1A-positive speckles become larger but less abundant already at time points at which cells exhibited no signs of degeneration (Figure 4B and Supplementary Figure S4B–D). We next addressed whether the Hub1 RNAi-dependent changes in nuclear speckle distribution are linked to defects in nuclear mRNA distribution. Indeed, fluorescence *in situ* hybridization (FISH) using poly (dT) probes revealed an moderate nuclear accumulation and enrichment of poly-adenylated RNA in nuclear speckles in Hub1 siRNA-treated cells compared with control cells (Figure 4B and Supplementary Figure S4B and D). The observed RNA accumulation was similar to cells in which splicing was repressed by the splicing inhibitor spliceostatin A (Kaida et al., 2007), repressing oligonucleotides that target U1 or U6 snRNAs (O'Keefe et al., 1994) or RNAi (Tanackovic and Krämer, 2005), further indicating that human cells lacking Hub1 suffer from defective pre-mRNA processing.

### Hub1 is crucial for splicing of certain introns

**Figure 5:**
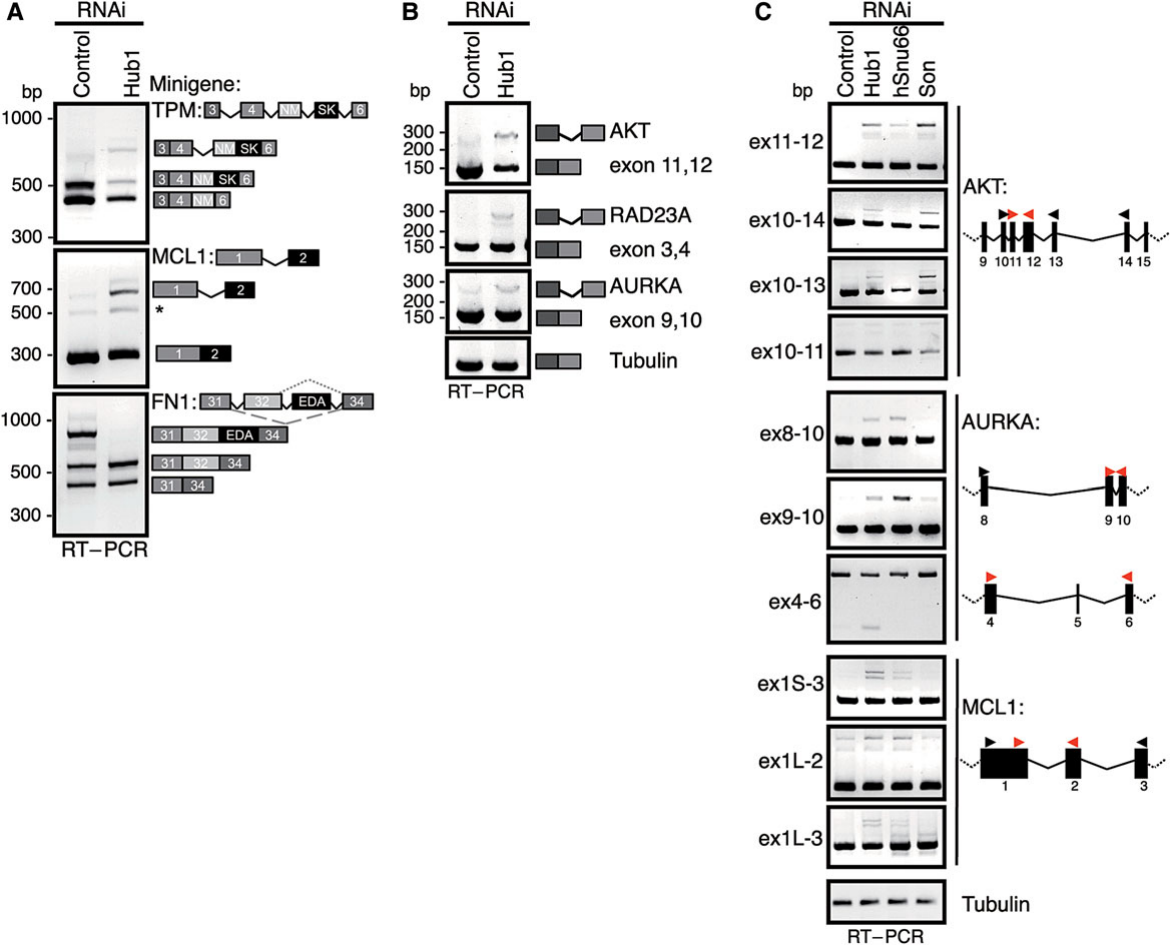
Hub1 is crucial for mRNA splicing of certain introns. (**A**) Alteration in alternative splicing of minigenes upon Hub1-depletion. Genomic fragments of tropomyosin 1α (*TPM*, exon 3–6), myeloid cell leukemia sequence 1 (*BCL2*-related) (*Mcl-1*, exon 1–2), or fibronectin 1 (*FN1*, exon 31–34 incl. *ED-A*) (see schematic exon-intron structure) expressed as minigenes in U2OS cells, and their mRNA products were analyzed by minigene-specific RT–PCR after Hub1 or control RNAi. (**B**) Detection of aberrant splicing of endogenous transcripts of v-akt murine thymoma viral oncogene homolog 1 (*AKT*), *RAD23* homolog A (*RAD23A*), and Aurora kinase A (*AURKA*) after Hub1 knockdown by intron-spanning RT–PCR. (**C**) Detailed characterization of splicing specificities dependent on Hub1 and comparison to splicing factors hSnu66 and Son. Splicing of Hub1-dependent introns and flanking exons in *AKT*, *AURKA*, and *MCL1* mRNAs after RNAi against Hub1, hSnu66, and Son in U2OS cells analyzed by gene-specific RT–PCR. Primer sets indicate Hub1-sensitive introns in the respective transcripts tested in RNAi experiments (red arrow heads), whereas mapping studies with PCR primers located in flanking sequences (black arrow head) detected no splicing alterations in neighboring exons/introns.

The strong phenotype associated with Hub1 depletion in human cells suggests that human Hub1 plays a much more fundamental cellular role than its *S. cerevisiae* counterpart. Reasonable models are that Hub1 is crucial in human cells either for general splicing, for splicing of certain pre-mRNAs or of particularly sensitive splicing reactions at suboptimal splice sites. To address splicing competence of Hub1-depleted tissue culture cells, we first analyzed splicing of model transcripts that are known to undergo alternative splicing in humans. To this end, we used a minigene approach (Stoss et al., 1999) by expressing genomic fragments of genes for fibronectin 1 (*FN1*, exon 31–34 including *ED-A* exon; Muro et al., 1999), tropomyosin 1α (*TPM*, exon 3–6; Graham et al., 1992) or myeloid cell leukemia sequence 1 (*MCL1*, exon 1–2; Bae et al., 2000) in U2OS or HeLa cells after treatment with Hub1 siRNA or control siRNA for 48 h, during which neither aberrant mitosis nor viability defects were detectable. Using minigene-specific primers, we measured splicing efficiency by RT–PCR of isolated total RNA. In this setup, Hub1 depletion resulted in different forms of alternative and constitutive splicing defects. In case of *FN1*, Hub1 depletion caused exon (*ED-A*) skipping, whereas intron retention coupled to moderately lower steady state mRNA levels was observed for *TPM* and *MCL1* (Figure 5A).

To determine whether Hub1 depletion also affects endogenous pre-mRNAs, we tested splicing of transcripts that are known to harbor weak splice sites by intron-spanning RT–PCR (Ahn et al., 2011). By these assays we observed different degrees of splicing defects for introns of the tested genes *AKT*, *RAD23A*, and *AURKA* (aurora kinase A), whereas tubulin pre-mRNA was spliced normally (Figure 5B). The observed defects could be directly attributed to RNAi-mediated Hub1 deficiency as indicated by complementation experiments using Hub1 siRNA-insensitive GFP-Hub1 cDNAs (Supplementary Figure S5A and B). Furthermore, since cDNA encoding a Hub1 variant deficient in hSnu66-binding (Hub1-D22A) complemented the splicing defects, yet perhaps not as well (Supplementary Figure S5A and B), direct binding of Hub1 to hSnu66 via the HIND element is apparently not strictly essential for Hub1-dependent splicing in humans.

We next compared the splicing defects linked to Hub1 depletion with splicing defects induced by depletion of the tri-snRNP protein hSnu66 or the SR protein Son, which supports splicing for a subset of human pre-mRNAs (Sharma et al., 2011). Whereas Hub1 and Son seemed important for efficient *AKT* splicing, Hub1 and hSnu66 (but apparently not Son) facilitated splicing of *MCL1* and *AURKA* (Figure 5C and Supplementary Figure S5D). Furthermore, detailed mapping studies of Hub1-sensitive transcripts by analyzing flanking exonic and intronic sequences revealed that Hub1 depletion caused splicing defects only for some introns, while other introns of the same pre-mRNAs were processed normally (Figure 5C). Notably, the observed splicing defects of these pre-mRNAs that occurred upon Hub1 depletion could also be verified using subcloned fragments in minigene splicing assays (Supplementary Figure S5C). This suggests that the effect of Hub1 on splicing does not require the native pre-mRNA or genomic chromatin, which can influence splice site selection.

To analyze the role of Hub1 for splicing events further we also undertook a genome-wide approach using a splicing-sensitive microarray. To this end, we performed exon-level expression profiling using the Affymetrix GeneChip Human Exon 1.0 ST array which allows the detection of alternative splicing events by multiple probes per exon along the entire length of a transcript (Clark et al., 2007). Bioinformatic analysis of the microarray core set identified >3000 altered splicing events in total (63707 probe sets tested) for distinct exons after Hub1 RNAi treatment compared with control RNAi treated U2OS cells (Geo Series Accession Number: GSE56878; Supplementary Table S3 and Figure S5E), further corroborating a substantial impact of Hub1 on a number of splice substrates. Thus, various lines of evidence indicate that Hub1 is not generally essential for splicing but crucial for only some particular splicing events leading to alternative splicing in human cells. Moreover and importantly, only certain splicing events for a given pre-mRNA are affected by Hub1-depletion, demonstrating that the requirement for Hub1 is not restricted to a certain class of mRNA species.

## Discussion

Posttranslational modification of proteins by members of the ubiquitin family affects numerous cellular functions. Due to the presence of modifier-conjugating and de-conjugating enzymes, these pathways often function as switches or reversibly control protein–protein interactions. Even though Hub1 is structurally very similar to canonical ubiquitin-like proteins (McNally et al., 2003; Ramelot et al., 2003), the mode of action of Hub1 is very different. Comprising solely the ubiquitin-fold and lacking N- or C-terminal extensions, Hub1 is only able to function through non-covalent interactions with other proteins (Luders et al., 2003; Yashiroda and Tanaka, 2004; Mishra et al., 2011). So far only binding of Hub1 to the spliceosome has been functionally confirmed and studied at the molecular level in yeast. In the spliceosome, a conserved binding partner of Hub1 is the tri-snRNP protein Snu66. Remarkably, the highly conserved Hub1-binding HIND element is found in Snu66 of yeast and vertebrates, but on Prp38, another tri-snRNP protein, in plants (Mishra et al., 2011). Moreover, in *Plasmodium*, both Snu66 and Prp38 homologs contain HIND elements capable in Hub1 binding. This phenomenon suggests that Hub1 binding is not crucial for its respective direct binding partner, but rather for the functional complex, the spliceosome. Since clear HIND elements are apparently not found in other proteins (Mishra et al., 2011), HIND elements seem to be specific for spliceosomal Hub1 recruitment and function.

With our new data on human Hub1 we are now in the position to compare the significance of Hub1 for different organisms and to identify the conserved and general role of Hub1 for pre-mRNA splicing. Based on protein–protein interaction studies and structural data, here we revealed the mechanism of binding of human Hub1 to Snu66 via the HIND element. Compared with non-covalent binding interfaces of ubiquitin and ubiquitin-like proteins, the Hub1–HIND structure highlights a unique mode of interaction and binding paradigm (Mishra et al., 2011). Several ubiquitin-binding domains (UBDs) have been identified and crystalized in complex with the ubiquitin protein. The vast majority of UBDs associate with ubiquitin via the hydrophobic area around I44 supported by L8 and V70 on sheets β3β4 (Dikic et al., 2009). In contrast, the Hub1–HIND interaction surface is located on the opposite side to the canonical UBD patch formed by helix α1 and sheets β1β2. Moreover, the Hub1–HIND complex also clearly differs from the non-covalent interaction of other ubiquitin-like modifiers like SUMO with its SIM (SUMO interaction motif; Song et al., 2005) and the Atg8 homolog LC3 with its interaction region (LIR) (Noda et al., 2008). We show that the mode of Hub1 binding to Snu66 is precisely conserved at the molecular level from *S. cerevisiae* to humans, while this specific interaction appears to be particularly relevant for *S. cerevisiae*. On the other hand, since only a relatively small portion of the surface of Hub1 is directly involved in Snu66 binding, it is conceivable that different surface areas of Hub1 make additional contacts with Snu66 or other proteins of the spliceosome.

In *S. cerevisiae*, for which it is assumed that the majority of original introns had been eliminated during evolution (Fink, 1987), Hub1 is not essential for viability and apparently functionally restricted to promote alternative splicing of *SRC1* pre-mRNA, which encodes a non-essential protein (Mishra et al., 2011). This currently only known Hub1-regulated target is also a yeast rarity since alternative splicing in *S. cerevisiae* is extremely rare. Notably, Hub1 becomes vital for *S. cerevisiae* if these cells are additionally partially defective in certain spliceosomal proteins like Prp8 (Mishra et al., 2011). From these lines of evidence we thus infer that splicing conducted by spliceosomes containing Hub1 is generally more robust by tolerating suboptimal splicing conditions.

Hub1 is essential for viability in human cells most likely because a large number of pre-mRNAs require Hub1 for optimal and correct splicing of its introns. Judging from our assays, a significant portion of splicing events in human cells entail Hub1 for normal (WT) splicing, although many other splicing events, even those from the same pre-mRNAs, were not affected by Hub1 depletion at all. Since the splice sites of the investigated introns that are sensitive to Hub1 show no obvious sequence similarity, Hub1 may act as a splicing qualifying factor for splicing events that are suboptimal for different reasons, e.g. due to pre-mRNA folding constrains (secondary structures) or the presence of bound proteins. Moreover, as indicated by our minigene and microarray data, Hub1-stimulated RNA processing affects a broad spectrum of splice events in various transcripts leading to alternative splicing in human cells. In striking contrast to canonical regulators of alternative splicing (e.g. SR proteins) that directly target pre-mRNAs by binding to crucial *cis*-regulatory elements in pre-mRNAs via their characteristic RNA-recognition motifs (Keren et al., 2010), Hub1 by being incorporated into the spliceosome appears to stimulate certain splicing events through altering the splicing machinery rather than by targeting specific RNA substrates.

The other cellular phenotypes we observed upon prolonged Hub1 depletion, like splicing speckle abnormalities, partial nuclear pre-mRNA retention, mitotic defects and apoptosis, are likely consequential effects caused by an accumulation of abnormally spliced pre-mRNAs and perhaps their potentially harmful products. Indeed, generation of aberrantly spliced transcripts can cause cellular stress, mis-regulation of various cellular pathways, cell cycle defects and potentially cancer (Venables, 2004). It is of note, however, that siRNA-mediated depletion of splicing factors caused mitotic defects only for a fraction (∼30%) of spliceosomal factors (Hofmann et al., 2010; Neumann et al., 2010) suggesting that spliceosomes may perhaps directly influence the cell cycle.

Based on our study, it seems reasonable to assume that at least some of the previously reported activities of Hub1 are linked to Hub1's role in splicing. However, as for the majority of proteins of large protein assemblies like the spliceosome, the ribosome or the nuclear pore, identifying the precise mechanistic explanation for Hub1's influence on splicing will be challenging. On the other hand, the conserved ability of Hub1 to support alternative splicing events indicates that mechanisms via modulating the spliceosome apparently complement more elaborate control systems for alternative splicing that are conducted by trans-acting factors that target the pre-mRNA.

## Materials and methods

### Yeast strains and plasmids

*S. cerevisiae* and *S. pombe* strains, complementation assays, immunoblot analysis and *SRC1* alternative splicing assays used in this study were described previously (Mishra et al., 2011). p415 ADH plasmid harboring coding sequences of *S. cerevisiae*, *S. pombe*, and human *HUB1* were used for complementation of *S. cerevisiae* mutants. pREP81 plasmid harboring coding sequences for *S. cerevisiae*, *S. pombe*, and human Hub1 were used for complementation of the *S. pombe* mutant.

### Plasmids and siRNA

Standard cloning techniques were used to generate mammalian expression constructs in pEGFP-N1 (Clontech) or p3xFlag-CMV-10 (Sigma-Aldrich) vectors. The cDNA clone for hSnu66 (SART1) was purchased from Origene, while the cDNA for human Hub1 (UBL5) was amplified by RT–PCR using total RNA from HeLa cells. Plasmids with point mutations or sequence deletions were constructed by site-directed mutagenesis using specific primers. Genomic fragments of fibronectin 1 (*FN1*), tropomyosin 1α (*TPM*), myeloid cell leukemia sequence 1 (*MCL1*), v-akt murine thymoma viral oncogene homolog 1 (*AKT*), and aurora kinase A (*AURKA*) for minigene constructs were amplified from genomic human DNA (U2OS) by PCR and subcloned into modified pUB6/V5 vectors (Invitrogen). For RNAi siRNA oligos were purchased from MWG and designed as 19- or 21-mer duplexes with 3′ TT-overhangs according to criteria previously described (Elbashir et al., 2001). siRNA duplexes targeting Hub1 in human cells were iHub1_1 GGAAGAAGGUCCGCGUUAA, iHub1_2 CAAGAUUGUCCUGAAGAAG, iHub1_3 AUAGAUGAGAAUCCUCAUC, iHub1_4 UGCAACACGGAUGAUACCA, iHub1_5 GGGAAGAAGGUCCGCGUUA, while siRNA CUAACAAACUCCGGGCAAA was used for hSnu66 knockdown. The GL2 siRNA targeting luciferase (Elbashir et al., 2001) CGUACGCGGAAUACUUCGA was used as knockdown control. RNAi of Son was performed with Silencer pre-designed siRNA (ID# 143161) from Ambion.

### Human cell lines and transfections

The established cell lines HeLa, U2OS, and HEK293 T were maintained at 37°C, 6% CO_2_ in DMEM (Invitrogen) supplemented with 10% fetal calf serum (FCS; Biochrom). HEK293T cells were transfected using the calcium phosphate precipitation technique as described previously (Bartke et al., 2004). Lipofectamine 2000 (Invitrogen) or Fugene HD (Roche) was used to transfect U2OS and HeLa cells. For RNA interference (RNAi) experiments, cells were transfected via electroporation with the Amaxa Nucleofector II system (Lonza) or in 6-well plates using RNAiMax (Invitrogen) with siRNA duplexes at a final concentration of 300 and 50 nM, respectively, according to the manufacturers' protocol. Due to highest knockdown efficiency Hub1 depletion and complementation experiments were performed using siRNA oligo iHub1_1 (Figures 3, 4B, 5 and Supplementary Figure S3–S5). However, repetition of knockdown experiments with iHub1_3 RNAi led to similar results. U2OS cells stably expressing GFP-Hub1 WT, GFP-Hub1 D22A, and free GFP were generated by several rounds of selection with 750 µg/ml G418 (Sigma-Aldrich) after lipofection. HeLa cells stably expressing histone variant H2B fused to green fluorescent protein (H2B-GFP) were established as described previously (Kanda et al., 1998) and examined in RNAi experiments using live cell imaging. Fluorescence-activated cell sorting was performed using the FACSAria cell sorter (Becton Dickinson) to further enrich and purify GFP-positive cells.

### Cell extracts and immunoprecipitation

Whole cell extracts were prepared by lysing human cells directly in SDS Laemmli buffer. For immunoprecipitation, cells were harvested, washed in ice-cold PBS and cell pellets were lysed in 5× pellet volumes of immunoprecipitation buffer (50 mM HEPES pH 7.2, 150 mM NaCl, 2 mM EDTA, 0.5% Triton X-100, 1 mM PMSF, and complete protease inhibitors (Roche)) at 4°C for 30 min with several passages through a 25 gauge needle attached to a syringe. After removal of cell debris by centrifugation (10 min, 16000 *g*, 4°C), cleared lysates were incubated with anti-FLAG M2 affinity gel (Sigma-Aldrich) or GFP trap (Chromotek) for 2 h at 4°C. The affinity matrix was washed four times with immunoprecipitation buffer and eluted in SDS Laemmli buffer for later analysis by SDS–PAGE and immunoblotting. For caspase activation assay, cytosolic extracts were prepared as described previously (Deveraux et al., 1999). Immunoblot quantification was performed using the ImageJ software.

### Flow cytometry and cell cycle synchronization

DNA histograms were obtained by flow cytometry analyses of PI-stained ethanol-fixed cells using standard protocols (propidium iodide 100 µg/ml (Sigma-Aldrich), RNase A 200 µg/ml (Sigma-Aldrich) in PBS). FITC-labeled Annexin V (Sigma-Aldrich) and PI (1 µg/ml) were used to detect the induction of apoptosis in unfixed RNAi-treated cells according to the manufacturer's protocol. Data were acquired on a FACSCalibur system with CELLQuest software (Becton Dickinson) and further analyzed with FlowJo software (Tree Star). HeLa cells were synchronized following standard double-thymidine block protocols using 2 mM thymidine.

### Live cell imaging, immunofluorescence, and FISH analysis

For standard immunofluorescence microscopy, U2OS cells were seeded and transfected on glass coverslips (Roth). Cells were washed twice with PBS and fixed in 3.7% fresh paraformaldehyde/PBS for 18 min at room temperature. After fixation residual formaldehyde was inactivated by quenching with PBS-glycine (30 mM) and cells were washed three times in PBS. Permeabilization of cells was performed using PBS-Triton X-100 0.4% (6 min), followed by three PBS-Tween (Tween 0.05%; PBS-T) washing steps and blocking in PBS-T with 2% BSA for 1 h at room temperature. Coverslips were incubated with primary antibody for 3 h (dilution 1:200 in blocking buffer) and then washed three times in PBS-T. After incubation with secondary antibody, cover slips were mounted using 4′,6-diamidino-2-phenylindole (DAPI)-containing mounting medium (Vectashield, Vector Labs).

For pre-extraction experiments cells were permeabilized in CSK buffer (100 mM NaCl, 300 mM sucrose, 10 mM PIPES pH 6.8) supplemented with 0.4% Triton X-100 and complete protease inhibitors (Roche) for 8 min at 4°C. After two gentle wash-out steps with detergent free CSK buffer, cells were fixed with 3.7% formaldehyde. The following antibody staining was performed according to the abovementioned standard protocol.

The RNA FISH method to visualize poly-adenylated mRNA using fluorescently labeled poly-(dT)_70_-TRITC (MWG) probes (Dias et al., 2010) was described previously (Tokunaga and Tani, 2008). Images of several optical sections were acquired on a Zeiss AxioImager Z1 microscope equipped with a CoolSNAP-HQ2 CCD camera (Photometrics) and AxioVision Rel. 4.7 software (Zeiss). In order to quantify the subcellular distribution of poly-adenylated mRNA in RNAi-treated cells images were further processed with CellProfiler analysis software (Carpenter et al., 2006). For cell segmentation nuclei were identified by DAPI counterstaining while cell body outlines were specified by phalloidin-FITC labeling (0.2 µg/ml; Sigma-Aldrich) of cellular actin networks to subsequently measure the integrated intensities of mRNA FISH signals in nuclear and cytoplasmic regions.

After RNAi transfection, HeLa H2B-GFP cells were seeded on 4 well µ-dishes (ibidi) and transferred into the BioStation IM live cell imaging system (Nikon) for fluorescence time-lapse microscopy. Images were acquired every 8 min over a time frame of 24−48 h with BioStation IM software and further processed by Photoshop (Adobe).

### Recombinant protein purification and crystallization

Vector (pET28a) harboring human *HUB1* was expressed in BL21-CodonPlus (DE3)-RIL cells (Stratagene) and the 6×His-tagged protein was purified by Ni-NTA agarose beads (Qiagen), followed by gel filtration on Superdex 75 (GE Healthcare) in PBS buffer. Proper folding of the protein was analyzed by 1D NMR spectrum recorded by a 600-MHz Bruker NMR spectrometer. The hSnu66 HIND peptide, comprising residues 117–135 of hSnu66, containing the sequence SLSIEETNKLRAKLGLKPL, was chemically synthesized.

For crystallization, purified Hub1 was mixed with the HIND peptide at a molar ratio of 1:3 and the complex was separated by gel filtration on Superdex 75 (GE Healthcare) in 10 mM Tris/HCl pH 8.0, 100 mM NaCl, and 5 mM β-mercaptoethanol. The complex was concentrated to 10–13 mg/ml and crystallized at 20°C, using the sitting drop vapor diffusion method. The 2–3 µl drops consisted of a 1:1 (*v/v*) mixture of protein solution and well solution. Crystals appeared after 3 days and grew to final size after 2 weeks of incubation. The best diffracting crystals of the human Hub1/HIND complex grew in 0.1 M Tris/HCl pH 9.0, 0.15 M sodium acetate, 20% (*w*/*v*) PEG 4000. Crystals were soaked in cryoprotection solution containing mother liquor supplemented with 30% glycerol and were snap frozen in liquid nitrogen.

### Data collection and structure determination

A high quality X-ray dataset up to 2.0 Å was collected at the Swiss Light Source beamline PXII at Paul Scherrer Institute (Villigen, Switzerland). The collected data were integrated, scaled and merged by XDS and XSCALE programs (Kabsch, 1993) in space group P21212.

The structure was determined by molecular replacement using the Molrep program from the CCP4 suite ccp (Collaborative Computational Project, Number 4, 1994) and the structure of the Sc Hub1/HINDII complex as a search model (PDB entry 3PLV). Refinement and model building were carried out with the REFMAC5 (Collaborative Computational Project, Number 4, 1994) and XtalViev/Xfit (McRee, 1999). The Arp/Warp (Lamzin and Wilson, 1993) program was used to add water molecules. Certain solvent exposed side-chains without clear electron density were removed from the model. Data collection and refinement statistics are shown in the Supplementary Table S1. The Ramachandran-plot distribution for residues in the structure was 95.4% in most favored regions, 3.4% in allowed regions, 1.2% in disallowed regions. All structural-model figures were generated by Pymol (http://www.pymol.org/).

### Antibodies

Antibodies used in this study were anti-α-tubulin (DM1A, Sigma-Aldrich), anti-Caspase-7 (C7, Cell Signaling), anti-Caspase-3 (8G10, Cell Signaling), anti-GFP (clone B-2, Santa Cruz and ab1218, Abcam), hnRNP I (sc-133667, Santa Cruz), anti-PRPF6 (sc-48786, Santa Cruz), anti-PRPF8 (ab87433, Abcam), anti-TAP (PAP) (Sigma-Aldrich), anti-hSnu66 (A301–423A, Bethyl), anti-SC35 (for immunofluorescence: ab11826, Abcam; for immunoblotting: #556363, BD Biosciences), anti-Son (HPA023535, Sigma-Aldrich), anti-2,2,7-trimethylguanosine (K121, Calbiochem), anti-U1A (ab55751, Abcam), anti-U2AF65 (ab37483, Abcam), anti-Sm antigen Y12 (ab3138, Abcam). For immunofluorescence Alexa Fluor 488- and Alexa Fluor 555-labeled secondary goat anti-mouse and donkey anti-rabbit/anti-mouse antibodies from Invitrogen were used. Hub1-specific antibodies against recombinant *S. cerevisiae* Hub1 and human Hub1, respectively, were affinity-purified from serum of immunized rabbits.

### RNA isolation, reverse transcription, and PCR

Total RNA was isolated from RNAi-transfected cells using the RNeasy kit (Qiagen) according to the manufacturer's protocol. Reverse transcription was performed with the Transcriptor First Strand cDNA Synthesis Kit with random hexamers or poly-(dT) primers (Roche), while subsequent transcript specific PCRs were conducted using PfuTurbo DNA polymerase (Aligent). PCR products were analyzed on 2%−2.5% ethidium bromide containing agarose gels. Gene-specific primer sequences are listed in Supplementary Table S2.

### Splicing-sensitive microarray analysis

Exon expression profiling was performed using the GeneChip Human Exon 1.0 ST Arrays (Affymetrix). Total RNA was isolated from U2OS cells 60 h after Hub1 or control RNAi transfection using the High Pure RNA Isolation Kit (Roche) according to the manufacturer's protocol. RNA samples of biological triplicates were labeled and hybridized to the splicing-sensitive microarray for subsequent bioinformatics analysis via Affymetrix Powertools (ATLAS Biolabs GmbH) as described previously (Rasche and Herwig, 2010). Relative expression profiles of individual probes after Hub1 or control knockdown in U2OS cells were processed using the ARH method, resulting in metascores based on Splice index (SI), *P*-value (*P*, log10), and arh-value (arh, >0.03 significant). For initial analysis CEL files were processed using AltAnalyze software (version 2.0) with core probe set filtering using DABG (detected above background *P*-value cutoff 0.05) and microarray analysis of differential splicing (MiDAS exon analysis parameters *P*-value cutoff 0.05) (Gardina et al., 2006; Xing et al., 2008). The resulting splice index corresponds to differential exon intensity levels in Hub1 knockdown samples compared with control cells after normalization.

## Supplementary material

Supplementary material is available at *Journal of Molecular Cell Biology* online.

## Funding

S.J. is supported by Max Planck Society, the Louis-Jeantet Foundation, Deutsche Forschungsgemeinschaft SPP1365, Centre for Integrated Protein Science Munich, Fonds der chemischen Industrie; T.A.H. by Max Planck Society, Marie Curie FP7-Reintegration-Grant within the 7th European Community Framework Program, and a project operated within the Foundation for Polish Science TEAM Program, co-financed by the EU European Regional Development Fund.

**Conflict of interest:** none declared.

## Supplementary Material

Supplementary Data
