# Supplementary material for: The conserved ubiquitin-like protein Hub1 plays a critical role in splicing in human cells
Source: J Mol Cell Biol. 2014 May 28;6(4):312–23. doi: 10.1093/jmcb/mju026 (PMC4141198; doi:10.1093/jmcb/mju026)
Supplement: Supplementary Data [file supp_6_4_312__index.html]

The conserved ubiquitin-like protein Hub1 plays a critical role in splicing in human cells — The conserved ubiquitin-like protein Hub1 plays a critical role in splicing in human cells — Supplementary Data 

# The conserved ubiquitin-like protein Hub1 plays a critical role in splicing in human cells

## Supplementary Data

Supplementary Data

**Files in this Data Supplement:**

- Supplementary Data - Pdf file
