# Supplementary material for: The conserved ubiquitin-like protein Hub1 plays a critical role in splicing in human cells
Source: J Mol Cell Biol. 2014 May 28;6(4):312–23. doi: 10.1093/jmcb/mju026 (PMC4141198; doi:10.1093/jmcb/mju026)
Supplement: Supplementary Data [file supp_mju026_mju026supp_data.pdf]

# Supplementary material

## The conserved ubiquitin-like protein Hub1 plays a critical role in splicing in human cells

Tim Ammon, Shravan Kumar Mishra, Kaja Kowalska, Grzegorz M. Popowicz, Tad A. Holak and Stefan Jentsch

**Supplementary Figures and Legends 1 - 5**

**Supplementary Tables 1 - 3**

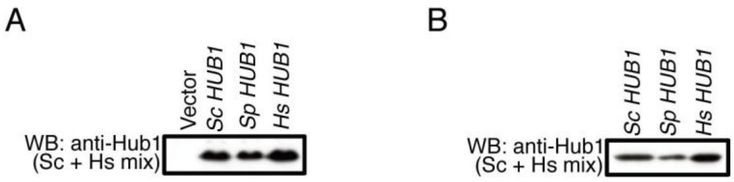


**Supplementary Figure S1**

(**A**) Protein expression control confirming equal levels of different Hub1 orthologs from *S. cerevisiae (Sc), S. pombe (Sp)* and *H. sapiens (Hs)* in *hub1Δ prp8* S. cerevisiae* strains detected by immunoblotting using anti-Hub1 antibodies (*Sc* and *Hs mix*). (**B**) Protein expression of different Hub1 orthologs (like in (**A**)) in *hub1Δ S. pombe* strains was monitored. Immunoblot was probed with anti-Hub1 antibodies.

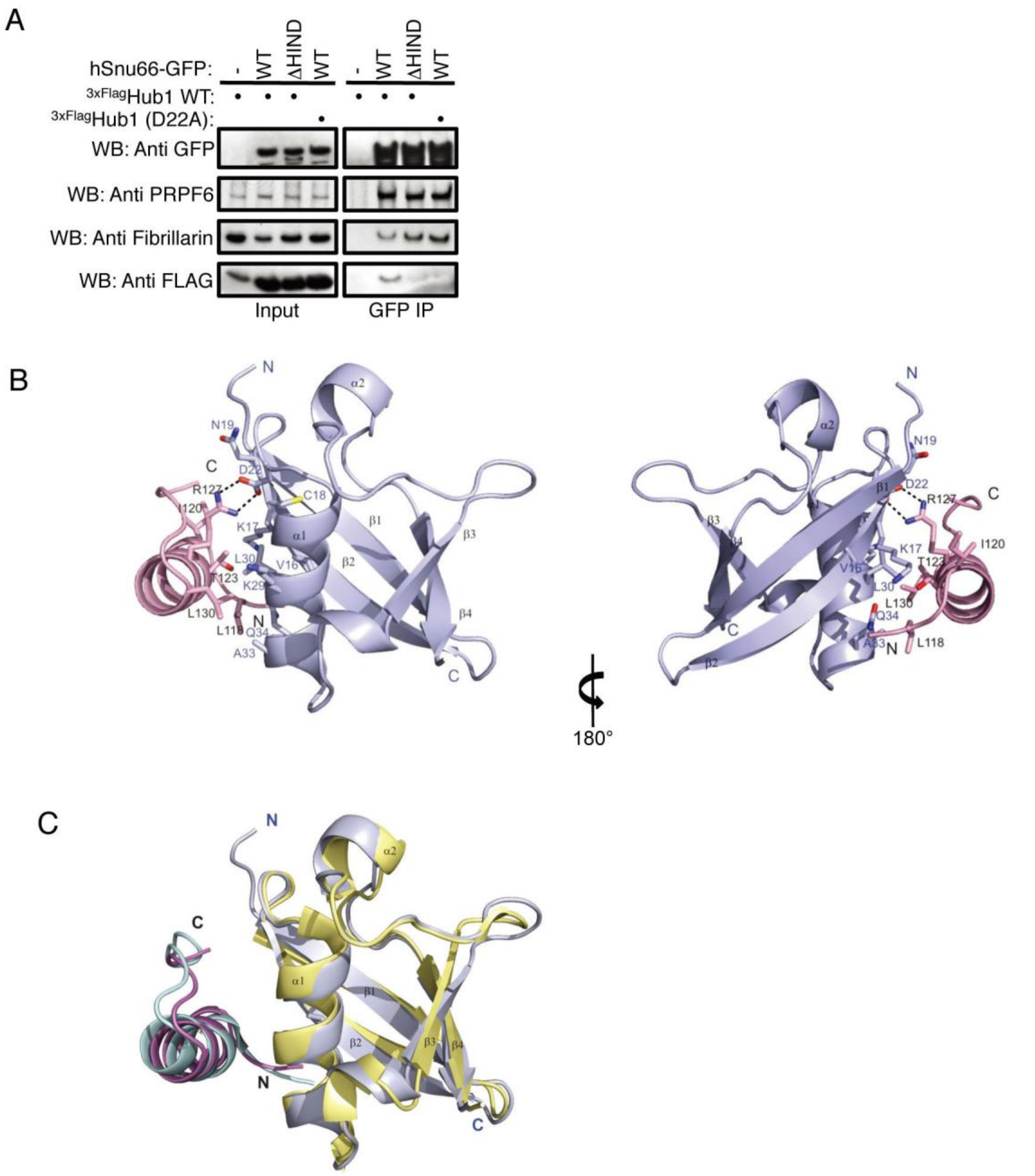


**Supplementary Figure S2**

(**A**) Co-immunoprecipitation of Hub1 with hSnu66 depends on the HIND interaction interface. GFP immunoprecipitation from HeLa cells after co-expression of GFP-tagged hSnu66 WT or the HIND-deletion mutant hSnu66 (hSnu66ΔHIND, deletion of aa 111-139) or free GFP with 3xFLAG-Hub1 WT or the hSnu66 binding-deficient mutant 3xFLAG-Hub1 (D22A). Immunoprecipitates were immunoblotted with anti-GFP and anti-FLAG antibodies with anti-PRPF6 (U4/U6.U5 tri-snRNP protein) and anti-Fibrillarin (nucleolar protein) serving as a positive control for hSnu66 co-immunoprecipitation. Note that in contrast to the loss of Hub1 binding, the interaction of hSnu66ΔHIND to PRPF6 or Fibrillarin was not affected by the deletion of the HIND.

(**B**) Two different orientations of the crystal structure showing human Hub1 (blue) in complex with the hSnu66 HIND peptide (pink) as a ribbon plot rotated by 180° along vertical axis. Amino acid residues involved in the formation of the binding interface are highlighted and labeled in stick representation. (**C**) Structural superposition of the human Hub1-HIND complex (blue, pink) with the *S. cerevisiae* ScHub1-ScHIND II (yellow, cyan, (Mishra et al., 2011)) shows high conservation on molecular level with a root mean squared deviation (RMSD) of 0.716 Å (PDB code 3PLV). The significant difference is found at the α2-β3 loop extending opposite of the Hub1-HIND interaction surface.

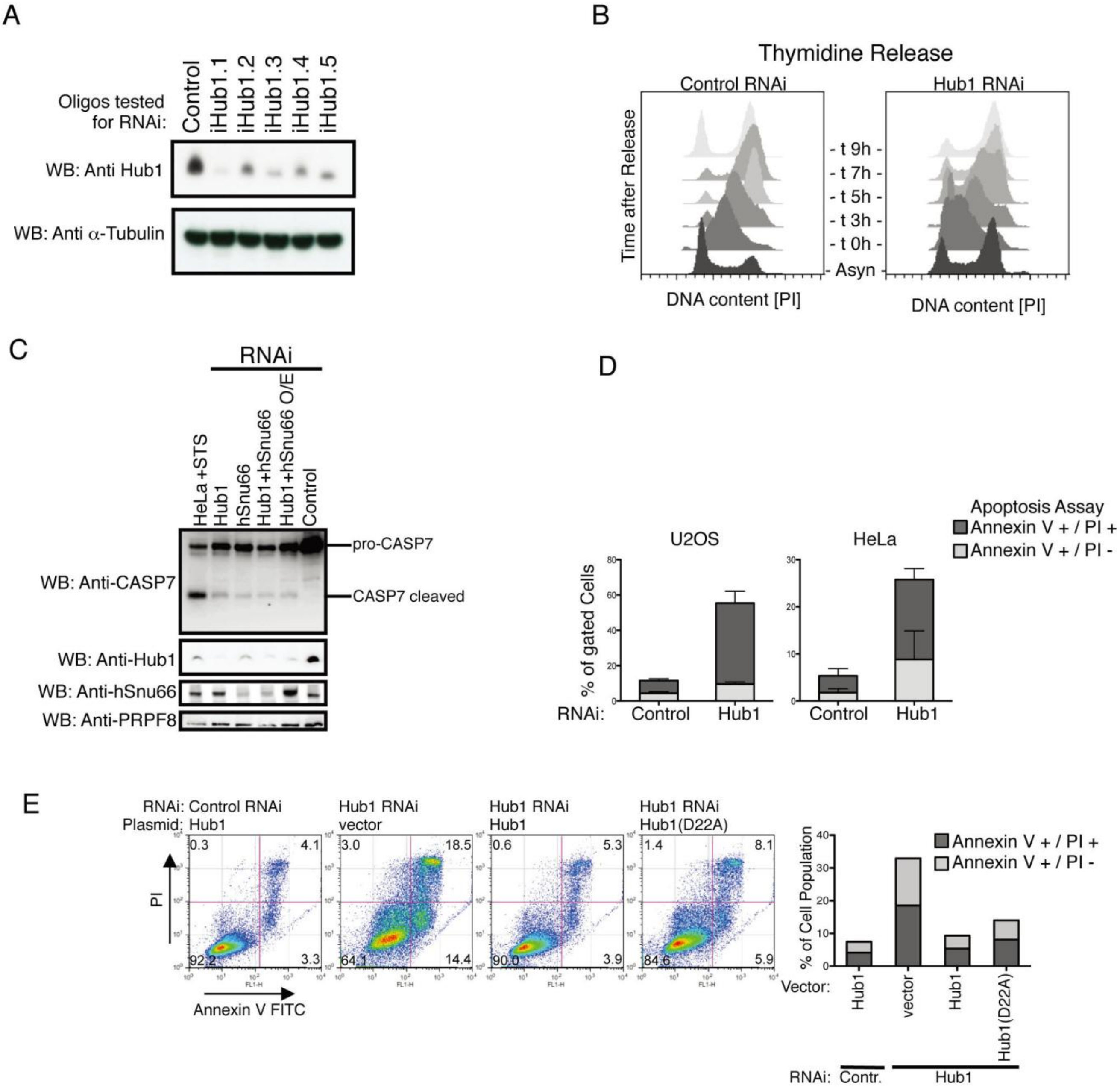


**Supplementary Figure S3**

(**A**) Verification of Hub1 knockdown efficiency of different siRNA oligos tested by immunoblotting with antibodies against human Hub1 and α-tubulin (loading control). (**B**) Cell cycle progression defects after Hub1 RNAi. After cell cycle synchronization by double-thymidine block the DNA contents of control or Hub1 RNAi treated cells were analyzed by flow cytometry at indicated time points after release into normal growth media. (**C**) Activation of the apoptotic cascade monitored by caspase 7 cleavage after RNAi. HeLa cells were transfected with RNAi targeting Hub1, hSnu66 or control and cotransfected with a plasmids expressing hSnu66-GFP, respectively.

HeLa cells treated with the protein kinase inhibitor staurosporine (STS, 2 µM for 5h) serve as a positive control. Immunoblots using antibodies against caspase 7 (detecting pro-caspase 7 and its activated (cleaved) form), Hub1, hSnu66 and loading control PRPF8 are shown. Similar results were obtained when assayed for caspase 3. (**D**) Flow cytometry-based detection of apoptosis induction in Hub1 knockdown cells. Human cells lines U2OS and HeLa, respectively, were transfected with control or Hub1-specific siRNA and cultivated for 72 hours. Subsequently, cells were harvested, stained and analyzed by flow cytometry, where the early apoptotic cell fraction was labeled by FITC-conjugated annexin v for externalized phosphatidylserine epitopes and late apoptotic cells became propidium iodide (PI)-positive due to loss of cell membrane integrity, respectively. Data represent mean and standard deviation of two independent experiments per cell line. (**E**) Annexin V / PI assay for Hub1 RNAi complementation experiments. After co-transfection of either control or Hub1-directed siRNAs together with constructs expressing siRNA-resistant Hub1 WT or Hub1 (D22A) mutant HeLa cells were analyzed by flow cytometry using FITC-conjugated annexin v and PI to identify apoptotic cells (see right panel for quantification of flow cytometry data).

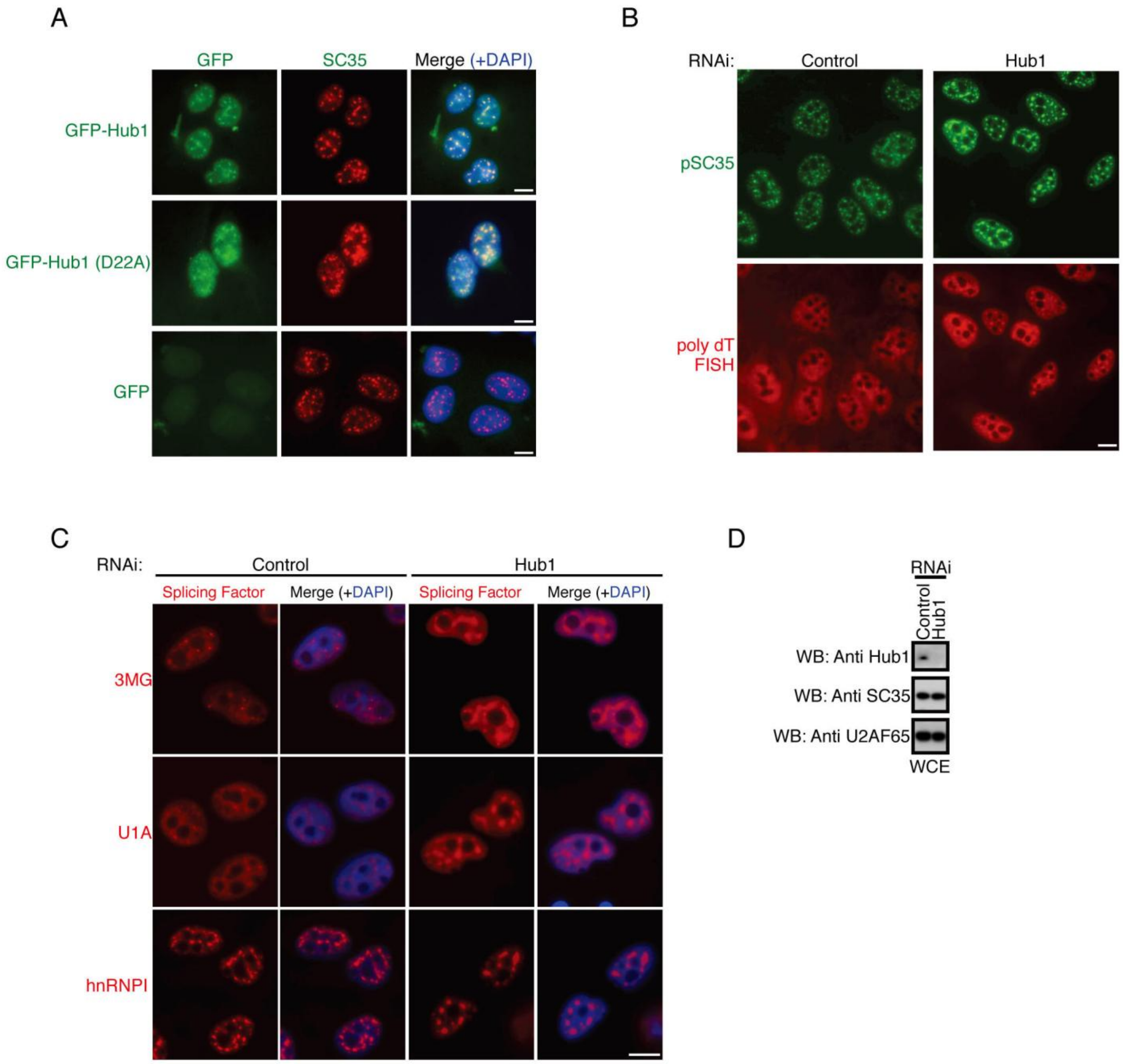


**Supplementary Figure S4**

(**A**) Co-localization studies in U2OS cells stably expressing GFP-Hub1 WT, hSnu66-binding mutant GFP-Hub1 (D22A) or free GFP (all green). Cells were pre-extracted, fixed and stained for nuclear speckle marker phospho-SC35 (red). Note that free GFP is washed out during pre-extraction, whereas GFP-Hub1 and GFP-Hub1 (D22A) are retained in nuclear speckles. Scale bar represents 10 µm. (**B**) Similar experiment as in Figure 4B showing a different set of cells for the visualization of poly-adenylated mRNA by FISH with fluorescently labeled poly-(dT)-TRITC probe co-stained for nuclear speckles with anti-SC35 antibodies in U2OS cells treated with

Hub1 or control RNAi (Scale bar 10 µm). (**C**) Changes in nuclear distribution of additional splicing-associated factors upon Hub1 knockdown. Immunostaining of 2,2,7-trimethylguanosine (3mG cap) of snRNAs, U1A or hnRNP I (all red) in control or Hub1 RNAi treated HeLa cells with nuclei counterstained with DAPI (blue). (Scale bars 10 µm). (**D**) Knockdown efficiency of Hub1-specific RNAi. Cells were transfected with control siRNA or oligos targeting Hub1 and whole cell extracts were tested by immunoblotting with specific antibodies against Hub1, SC35 and U2AF65 to verify protein *in vivo* depletion (referring to Figure 4**B**).

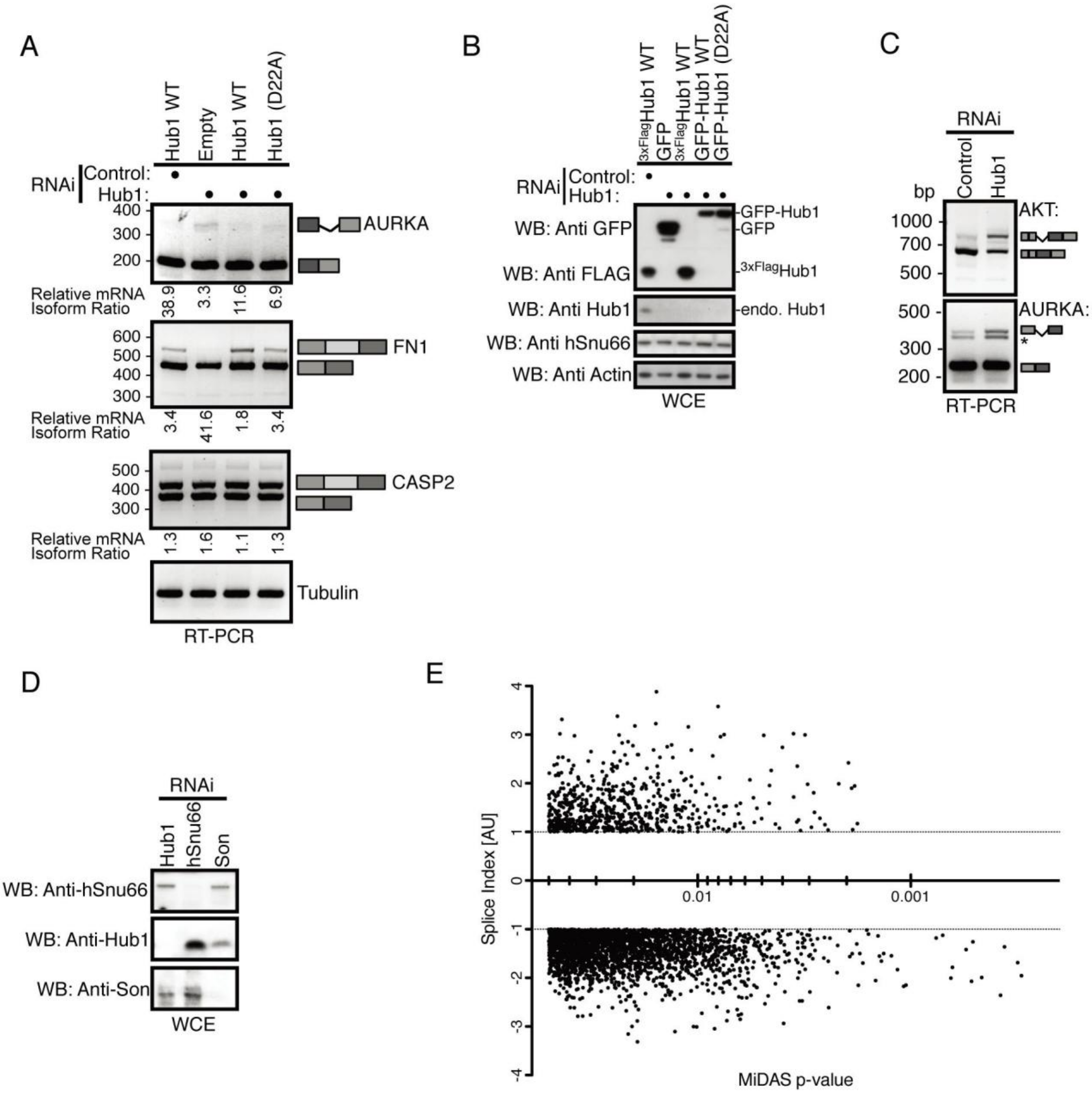


**Supplementary Figure S5**

(**A**) Complementation assay of splicing defects after *in vivo* depletion of Hub1. U2OS cells were co-transfected with either control or Hub1 siRNA and mammalian expression constructs encoding RNAi-resistant GFP-Hub1 WT, GFP-Hub1 (D22A) or free GFP (empty). For minigene analyses plasmids expressing genomic fragments of *FN1* or *CASP2* (as a Hub1-independent negative control) were introduced into knockdown cells. After 48h cells were harvested, total RNA was isolated and mRNAs were subjected to intron-spanning RT-PCR. Tubulin served as loading control. (**C**) Hub1 protein levels in RNAi complementation assays. After co-transfection of control or Hub1 siRNA with constructs expressing RNAi-resistant GFP-Hub1 WT, GFP-Hub1

(D22A), 3XFLAG-Hub1 WT or free GFP whole cell extracts were tested by immunoblotting with specific antibodies against endogenous Hub1, RNAi-resistant tagged Hub1 (anti-FLAG or anti-GFP) while hSnu66 and Action serve as loading controls. (**C**) Aberrant splicing of *AKT* and *AURKA* after Hub1 knockdown in RT-PCR based minigene assays. Genomic fragments containing alternative spliced exons of *AKT* (exon 9-12) and *AURKA* (exon 8-10) were expressed as minigenes in U2OS cells and their gene products were analyzed by minigene-specific RT-PCR after Hub1 or control RNAi. (**D**) Knockdown efficiency of splicing factor-specific RNAi. Cells were transfected with siRNA oligos targeting Hub1, hSnu66 or Son and whole cell extracts were tested by immunoblotting with specific antibodies against Hub1, hSnu66 and Son to verify protein *in vivo* depletion (referring to Figure 5**C**). (**E**) Splicing-sensitive microarray analysis revealed global alterations in alternative splicing patterns in U2OS cells upon Hub1 depletion. Total RNA was isolated from U2OS cells 60h after Hub1 or control RNAi transfection. Three biological replicates were labeled and hybridized to the Affymetrix Human Exon 1.0 ST microarray for subsequent bioinformatics analysis (ATLAS Biolabs GmbH, Germany). Genome-wide exon expression profiling detected 3226 altered splice events (out of 63707 microarray core probe sets) with differential exon expression intensities (cut off Splice Index SI $<-1$ and Si $>1$) in Hub1-knockdown datasets after probe set filtering using DABG (detected above background p value cutoff 0.05) and microarray analysis of differential splicing (MiDAS exon analysis parameters p value cutoff 0.05) using AltAnalyzer software.

## Supplementary Table S1

Data collection and refinement statistics for the human Hub1-HIND complex.

| | |
|---|---|
| Space group | P $2_1 2_1 2$ |
| Cell dimensions | |
| *a, b, c (Å)* | 87.51, 103.63, 67 |
| *α, β, γ (°)* | 90, 90, 90 |
| Resolution (Å) | 50 - 2.0 (2.1 - 2.0)* |
| Completeness (%) | 99.7 (99.8) |
| $R_{merge}$ | 6.4 (34.8) |
| I/σ(I) | 21.9 (4.48) |
| Redundancy | 7.32 (7.2) |
| Refinement | |
| No. of reflections | 35644 |
| Resolution (Å) | 20 - 2.0 |
| $R_{work}$ / $R_{free}$ (%) | 21.5 / 26.6 |
| No. atoms | |
| Protein | 5376 |
| Water | 206 |
| Overall B (Å$^2$) | 30.6 |
| r. m. s. deviations | |
| Bond length (Å) | 0.01 |
| Bond angles ($^o$) | 1.43 |

*Values in parentheses are for highest-resolution shell.

**Supplementary Table S2**

Primer used for transcript specific PCR analysis after revere transcription of isolated total RNA.

| Gene | Oligo | Sequence |
|---|---|---|
| ***AKT1*** | AKT1 Ex12 rev | GATGGTGATCATCTGGGCCGTGAACTCC |
| ***AKT1*** | AKT1 Ex11 rev | GTGCTGCCACACGATACCGGCAAAGAA |
| ***AKT1*** | AKT1 Ex8 for | GTTCTTCCACCTGTCCCGGGAGCGTGT |
| ***AKT1*** | AKT1 Ex6 rev | GAGGAAGGGGTGCCTGGAGTTCTGCA |
| ***AURKA*** | AURKA Ex10 far rev | CTAGCTGATTCTTTGTTTTGGCAATTTG |
| ***AURKA*** | AURKA Ex8 for | TGCCCTGTCTTACTGTCATTCGAAGAG |
| ***AURKA*** | AURKA Ex 4 for | CAGTCACAAGCCGGTTCAGAATCAGAA |
| ***AURKA*** | AURKA Ex 6 rev | TTCTCTTCTGAGCTGATGCTCCACTCC |
| ***Mcl1*** | TA.Mcl1-for | GAGGAGGAGGAGGACGAGTT |
| ***Mcl1*** | TA.Mcl1-Rev | ACCAGCTCCTACTCCAGCAA |
| ***Mcl1*** | Mcl1 Ex 1 for | GGCAGTCGCTGGAGATTATCTCTCG |
| ***Mcl1*** | Mcl1 Ex 2AS rev | GAGAGTCACAATCCTGCCCCAGTTTG |
| ***Mcl1*** | Mcl1 Ex 3 rev | CTACTCCAGCAACACCTGCAAAAGC |
| **pUB6-minigeneFor** | pUBSplicRepfor | TGTACCAGACTACGCTGGCCGGGAGAACC |
| **pUB6-minigeneRev** | pUBSplicReprev | GGTACGCGTAGAATCGAGACCGAGGAGAGGG |
| ***B-ACTIN*** | B-ACTINfor | GCCCCCCTGAACCCCAAGGCCAACCG |
| ***B-ACTIN*** | B-ACTINrev | GAAGTCCAGGGCGACGTAGCACAG |
| ***TUBA1*** | TUBA1B 23for | CCGGGCTGTGTTTGTAGACT |
| ***TUBA1*** | TUBA1B 23rev | GATCTCCTTGCCAATGGTGT |
| ***AKT1*** | AKT1 1112for | ACAAGGACGGGCACATTAAG |
| ***AKT1*** | AKT1 1112rev | ACCGCACATCATCTCGTACA |
| ***RAD23A*** | RAD23A 34for | TGCCCCAGAGTCCTCTACAT |
| ***RAD23A*** | RAD23A 34rev | GCTGCTACCTGAAGAGGGAAC |
| ***AURKA*** | AURKA 910for | AATGATTGAAGGTCGGATGC |
| ***AURKA*** | AURKA 910re | TCTGGCTGGGATTATGCTTC |

Last four primer pairs for *TUBA1*, *AKT1*, *RAD23A* and *AURKA* have been used in a previous study (Ahn et al., 2011).

Primers listed below were used to amplify genomic fragments in order to generate minigene constructs by cloning PCR products via Fse1/Asc1 into modified pUB6/V5 vectors (Invitrogen).

| Minigene | Oligo | Sequence |
|---|---|---|
| ***Fibronectin 1*** | FN1_EDA_For | GCGGCCGGCCAATTGACAAACCATCCCAGATG |
| ***Fibronectin 1*** | FN1_EDA_Rev | AGAGGCGCGCCATAAGTCCTGATACAACCACG |
| ***Mcl1*** | Mcl1-1-2-3_For | GCGGCCGGCCCATGTTTGGCCTCAAAAGAAAC |
| ***Mcl1*** | Mcl1-1-2-3_Rev | GAGGCGCGCCAGCCTCTTTGTTTAACTAGCCA |
| ***AKT1*** | Akt_9-12_For | GCGGCCGGCCGCTGTTCTTCCACCTGTCCCGG |
| ***AKT1*** | Akt_9-12_Rev | AGAGGCGCGCCTCTTCTCGTACACGTGCTGCC |
| ***Tropomyosin 1*** | TPM_3-6_For | GCGGCCGGCCAGGCATGAAAGTCATTGAGAGTCG |
| ***Tropomyosin 1*** | TPM_3-6_Rev | AGAGGCGCGCCCTCCTTCAGCTTGTCGGAAAGG |
| ***AURKA*** | AURKA_8-10_For | GCGGCCGGCCGTATATAACAGAATTGGCAAAT |
| ***AURKA*** | AURKA_8-10_Rev | GAGGCGCGCCAAGACTGTTTGCTAGCTGATTC |
| ***Casp2*** | Casp2_Ex8-10 For | GCGGCCGGCCCCAAGAGGTTTTTCAGCTCTTT |
| ***Casp2*** | Casp2_Ex8-10 Rev | GAGGCGCGCCCTTTGAGGCAGGCATAGCCGCA |

# Supplementary Table S3

Ranking of top 100 high confidence hits with significant splicing alterations upon Hub1 depletion. Bioinformatics analysis of exon-specific Affymetrix Human Exon 1.0 ST microarray data via Affymetrix Powertools at ATLAS Biolabs GmbH. Relative expression profiles of individual probes after Hub1 or control knockdown in U2OS cells were processed using the ARH method (Rasche and Herwig, 2010), resulting in metascores based on Splice index (SI), p-value (P, log10) and arh-value (arh, > 0.03 significant). The metascores are weighted and ranked according to unfiltered (0), filtered for cross-hybridization and expression constrains (1) and intensity > mean intensity filter (2). SI values and gene annotations of affected transcripts are shown.

| rank | transcript | ARH.SI0 | ARH.SI1 | ARH.SI2 | ARH.P0 | ARH.P1 | ARH.P2 | ARH.arh0 | ARH.arh1 | ARH.arh2 | gene | chr | strand | min_beg | max_end |
|---|---|---|---|---|---|---|---|---|---|---|---|---|---|---|---|
| | weight | 1 | 2 | 3 | 1 | 2 | 3 | 1 | 2 | 3 | | | | | |
| 1 | 2501835 | 10.7232056 | 10.581845 | 2.120701 | 8.6525852 | 8.607948 | 4.051119 | 12.55874231 | 12.1359193 | 0.3590293 | DPP10 | chr2 | + | 114916411 | 116318091 |
| 2 | 3893910 | 11.7129755 | 11.989843 | 2.350631 | 8.3339445 | 8.140057 | 4.813443 | 9.8347101 | 8.4749532 | 0.6521807 | TCEA2 | chr20 | + | 62159056 | 62174079 |
| 3 | 3185643 | 11.9817772 | 12.072458 | 2.357272 | 8.0817077 | 8.165058 | 3.67568 | 8.10376302 | 8.6391489 | 0.2666785 | RGS3 | chr9 | + | 115264200 | 115399643 |
| 4 | 3551485 | 10.3275402 | 10.337317 | 2.018001 | 8.1754139 | 8.160002 | 3.647695 | 8.70808766 | 8.6056847 | 0.2608011 | EML1 | chr14 | + | 99329506 | 99477554 |
| 5 | 3913018 | 8.0671691 | 8.020611 | 2.383467 | 8.184862 | 8.209734 | 4.087277 | 8.77146417 | 8.9405088 | 0.3694076 | LAMA5 | chr20 | - | 60317536 | 60375742 |
| 6 | 2519577 | 10.852828 | 3.996338 | 3.849249 | 8.3306295 | 5.493744 | 5.587729 | 9.80972259 | 1.1056498 | 1.1890146 | COL3A1 | chr2 | + | 189547347 | 189585676 |
| 7 | 2463227 | 8.3697921 | 5.031964 | 1.772492 | 8.1300703 | 6.676215 | 3.779859 | 8.41023804 | 2.7522442 | 0.2897009 | RGS7 | chr1 | - | 239005588 | 239587058 |
| 8 | 3917938 | 10.6509762 | 10.716544 | 2.018988 | 7.7170268 | 7.71529 | 3.670417 | 6.12484239 | 6.1166807 | 0.2655635 | HUNK | chr21 | + | 32167531 | 32297463 |
| 9 | 3354879 | 10.0105063 | 10.171977 | 1.841488 | 7.4196304 | 7.481308 | 4.086262 | 4.87411399 | 5.1106059 | 0.3691123 | HYLS1 | chr11 | + | 125258999 | 125275737 |
| 10 | 3634852 | 0 | 2.80488 | 2.911799 | 9.34349 | 5.123141 | 5.292353 | 21.33625907 | 0.8296814 | 0.9460143 | RASGRF1 | chr15 | - | 77041549 | 77170139 |
| 11 | 2461473 | 3.1208567 | 3.077959 | 2.432514 | 8.0993754 | 4.386554 | 4.116274 | 8.21440866 | 0.4673056 | 0.3779409 | TARBP1 | chr1 | - | 232593783 | 232681450 |
| 12 | 2566586 | 8.6188824 | 2.04957 | 1.832663 | 8.9850155 | 4.101378 | 4.470902 | 16.20703393 | 0.3735336 | 0.4992032 | TSGA10 | chr2 | - | 98980254 | 99124404 |
| 13 | 2582701 | 9.0204614 | 2.089432 | 2.499915 | 8.7200494 | 4.830687 | 4.674334 | 13.22589913 | 0.6609954 | 0.585178 | CCDC148 | chr2 | - | 158736743 | 159021459 |
| 14 | 3302805 | 11.4921363 | 2.546624 | 2.689921 | 8.3287854 | 3.909559 | 4.51817 | 9.79585017 | 0.3210553 | 0.5180009 | HPS1 | chr10 | - | 100165998 | 100196611 |
| 15 | 3422144 | 5.8659622 | 5.854478 | 1.901704 | 7.297323 | 7.37365 | 3.577812 | 4.43696271 | 4.7049436 | 0.246663 | LGR5 | chr12 | + | 70120128 | 70264733 |
| 16 | 3273251 | 4.5193368 | 4.742935 | 4.793449 | 6.1046848 | 6.191633 | 6.733953 | 1.77222167 | 1.8950787 | 2.877266 | DIP2C | chr10 | - | 312652 | 725603 |
| 17 | 2585400 | 5.8321041 | 3.46545 | 3.439895 | 7.1515138 | 5.581012 | 5.575142 | 3.96663759 | 1.1828548 | 1.1774986 | SCN9A | chr2 | - | 166763252 | 166876485 |
| 18 | 3657041 | 0 | 3.575804 | 3.786776 | 7.8409951 | 5.312817 | 5.719377 | 6.73649529 | 0.9611337 | 1.3163609 | ITGAX | chr16 | + | 31274023 | 31301804 |
| 19 | 2475116 | 5.9241822 | 2.289401 | 2.70111 | 7.5874672 | 4.499316 | 5.023979 | 5.54478017 | 0.5104214 | 0.7682006 | PLB1 | chr2 | + | 28678289 | 28719575 |
| 20 | 2755053 | 3.4151711 | 3.506007 | 3.586959 | 5.9971918 | 5.978986 | 6.261171 | 1.63122896 | 1.6084789 | 1.9994032 | CYP4V2 | chr4 | + | 187350017 | 187371582 |
| 21 | 3435515 | 3.5457711 | 3.790841 | 3.158279 | 5.9692412 | 6.309797 | 5.948787 | 1.59643174 | 2.0757299 | 1.5714353 | CCDC62 | chr12 | + | 121825090 | 121877866 |
| 22 | 3758317 | 4.1651517 | 4.165152 | 4.371097 | 5.6489255 | 5.648926 | 5.876556 | 1.24661528 | 1.2466153 | 1.4862351 | BRCA1 | chr17 | - | 38450837 | 38530785 |
| 23 | 2960955 | 4.0464658 | 3.856279 | 3.684563 | 5.7922626 | 6.015758 | 5.809962 | 1.39259326 | 1.6547585 | 1.4117565 | SLC17A5 | chr6 | - | 74359988 | 74420654 |
| 24 | 3898355 | 2.6013589 | 2.480013 | 2.461741 | 5.9033047 | 6.505772 | 6.539214 | 1.5172382 | 2.4138794 | 2.4768309 | FLRT3 | chr20 | - | 14252688 | 14266267 |
| 25 | 2759654 | 3.7338497 | 3.76364 | 3.721303 | 5.6140359 | 5.666395 | 5.889923 | 1.21344495 | 1.2635603 | 1.5016485 | ABLIM2 | chr4 | - | 8018189 | 8211389 |
| 26 | 2876608 | 1.4945056 | 3.503434 | 2.509572 | 3.9891621 | 6.724114 | 6.913396 | 0.34190102 | 2.8555686 | 3.303092 | CXCL14 | chr5 | - | 134934363 | 134942679 |
| 27 | 3898796 | 4.3113492 | 4.49917 | 2.214406 | 6.0671513 | 6.129095 | 3.444757 | 1.72166556 | 1.8058906 | 0.2217505 | KIF16B | chr20 | - | 16201510 | 16502068 |
| 28 | 3820161 | 3.0157962 | 3.015796 | 2.395702 | 5.3909034 | 5.390903 | 6.394102 | 1.02105554 | 1.0210555 | 2.2149948 | UBL5 | chr19 | + | 9799673 | 9801762 |
| 29 | 3108526 | 3.2844283 | 3.223018 | 3.033262 | 5.3942827 | 5.410787 | 5.678193 | 1.02373062 | 1.0368951 | 1.2751333 | MATN2 | chr8 | + | 98950579 | 99117487 |
| 30 | 3336277 | 3.5386987 | 3.582743 | 2.249787 | 6.1170585 | 5.856222 | 4.170249 | 1.78920989 | 1.4630891 | 0.3943393 | BBS1 | chr11 | + | 66034707 | 66057195 |
| 31 | 3632806 | 3.9103363 | 3.580188 | 2.379533 | 5.7132657 | 5.567839 | 4.700169 | 1.31016101 | 1.1708673 | 0.5970872 | STRA6 | chr15 | - | 72258934 | 72282168 |
| 32 | 3946380 | 3.2571499 | 3.224066 | 3.461071 | 5.1376619 | 5.026861 | 5.796719 | 0.83908349 | 0.7699224 | 1.3973938 | SGSM3 | chr22 | + | 39126663 | 39136036 |
| 33 | 2494064 | 3.4709614 | 3.470961 | 1.920732 | 6.0929172 | 6.092917 | 3.879759 | 1.75621444 | 1.7562144 | 0.313573 | LOC729234 FAHD2A FAHD2B | chr2 | + | 95432175 | 95442597 |
| 34 | 2404693 | 4.1092645 | 4.07012 | 1.829573 | 5.8415193 | 5.895639 | 3.408313 | 1.44657679 | 1.5082886 | 0.2153586 | BAI2 | chr1 | - | 31965309 | 32002184 |
| 35 | 3430959 | 3.5710411 | 3.076628 | 3.117127 | 5.3857137 | 5.53269 | 4.777939 | 1.01696085 | 1.1394682 | 0.6343951 | ACACB | chr12 | + | 108038798 | 108188532 |
| 36 | 3669650 | 3.209434 | 3.206321 | 1.754068 | 6.136146 | 5.880192 | 3.775031 | 1.81573386 | 1.4904129 | 0.2885928 | WWOX | chr16 | + | 76691181 | 77804044 |
| 37 | 3463056 | 2.2036707 | 2.769352 | 2.460489 | 5.1986282 | 5.298222 | 6.034637 | 0.87971937 | 0.9503261 | 1.6790314 | CSRP2 | chr12 | - | 75776692 | 75796930 |
| 38 | 2388525 | 3.2928082 | 3.255857 | 3.397471 | 4.9336404 | 4.94979 | 5.08752 | 0.71611677 | 0.7251649 | 0.8070567 | SDCCAG8 | chr1 | + | 241486135 | 241729709 |
| 39 | 3192580 | 3.1522646 | 2.514361 | 2.178652 | 5.1104249 | 5.832582 | 5.207671 | 0.82153395 | 1.4366296 | 0.8859099 | C9orf9 | chr9 | + | 134743578 | 134755237 |
| 40 | 2566848 | 2.6294392 | 2.666527 | 2.544907 | 4.9276776 | 5.110505 | 5.998505 | 0.7128042 | 0.8215853 | 1.6328818 | AFF3 | chr2 | - | 100087296 | 99991815 |
| 41 | 3278198 | 3.3523227 | 3.352323 | 2.321148 | 5.2160026 | 5.216003 | 4.953448 | 0.89165194 | 0.8916519 | 0.7272299 | PHYH | chr10 | - | 13359822 | 13382033 |
| 42 | 3174121 | 3.0524206 | 3.052421 | 3.257811 | 4.8504475 | 4.850448 | 5.195682 | 0.67124043 | 0.6712404 | 0.8777113 | MAMDC2 | chr9 | + | 71848392 | 72031670 |
| 43 | 3798829 | 3.1437151 | 3.143715 | 3.162063 | 5.0531895 | 5.053189 | 4.661344 | 0.78582843 | 0.7858284 | 0.5792782 | FAM38B2 | chr18 | - | 10747970 | 10777182 |
| 44 | 3696524 | 3.6035625 | 3.358578 | 1.743709 | 5.3216598 | 5.40053 | 4.375084 | 0.96774073 | 1.0286943 | 0.4631251 | PDF COG8 | chr16 | - | 67920122 | 67931014 |
| 45 | 3545634 | 2.3056701 | 2.819523 | 2.681055 | 4.5510024 | 4.435085 | 6.291083 | 0.53146461 | 0.4854062 | 2.0460177 | NRXN3 | chr14 | + | 78181216 | 79400194 |
| 46 | 2837232 | 3.0430718 | 2.94491 | 2.927819 | 5.0601711 | 4.871203 | 4.936612 | 0.79010035 | 0.6821699 | 0.7177731 | ITK | chr5 | + | 156540499 | 156614549 |
| 47 | 3059464 | 2.9206917 | 2.8936 | 3.035471 | 4.6150278 | 4.742255 | 5.400495 | 0.55871475 | 0.6170003 | 1.0286666 | SEMA3A | chr7 | - | 83428631 | 83661808 |
| 48 | 3150844 | 2.5643724 | 2.793296 | 3.042456 | 4.9651676 | 4.938614 | 5.14418 | 0.73388523 | 0.7188912 | 0.8433381 | SNTB1 | chr8 | - | 121619523 | 121893910 |
| 49 | 2890660 | 3.0300338 | 3.051791 | 3.54343 | 4.8153644 | 4.512698 | 4.854165 | 0.65315702 | 0.5157899 | 0.6731852 | GFPT2 | chr5 | - | 179660336 | 179712888 |
| 50 | 3770052 | 3.1013113 | 3.221057 | 3.215222 | 4.5820744 | 4.738903 | 4.864487 | 0.54452232 | 0.6153908 | 0.6786143 | SDK2 | chr17 | - | 68846024 | 68945556 |
| 51 | 3011911 | 0 | 2.359055 | 1.991251 | 7.5497671 | 4.045582 | 3.499442 | 5.38653121 | 0.3574651 | 0.2316829 | C7orf63 | chr7 | + | 89712489 | 89777485 |
| 52 | 3484497 | 3.259253 | 3.1552 | 3.33233 | 4.7474531 | 4.274253 | 4.903427 | 0.61950472 | 0.4279151 | 0.6994872 | FRY | chr13 | + | 31503933 | 31768116 |
| 53 | 2584258 | 2.8744953 | 2.85656 | 2.375771 | 5.085786 | 5.156842 | 4.730173 | 0.8059711 | 0.8516632 | 0.6112177 | KCNH7 | chr2 | - | 162936266 | 163403440 |
| 54 | 3637818 | 2.6413211 | 2.641321 | 1.800339 | 5.72059 | 5.72059 | 3.728118 | 1.31759555 | 1.3175955 | 0.278037 | NTRK3 | chr15 | - | 86221226 | 86600371 |
| 55 | 2358393 | 2.7300238 | 2.845384 | 2.47332 | 4.7912999 | 5.068634 | 4.937601 | 0.64103137 | 0.7953094 | 0.7183253 | ADAMTSL4 C1orf138 | chr1 | + | 148788522 | 148799763 |
| 56 | 3953556 | 1.9043424 | 3.10786 | 3.150803 | 4.0427097 | 5.469203 | 5.032276 | 0.35665647 | 1.084853 | 0.7731679 | KLHL22 | chr22 | - | 19125833 | 19180083 |
| 57 | 3349453 | 3.060273 | 3.369369 | 2.562768 | 5.0658868 | 5.150265 | 3.639233 | 0.7936148 | 0.847329 | 0.2590485 | TTC12 | chr11 | + | 112692202 | 112744393 |
| 58 | 2745899 | 2.8074192 | 2.876065 | 3.250158 | 4.6436603 | 4.654636 | 4.681673 | 0.57134074 | 0.5762547 | 0.5885369 | HHIP | chr4 | + | 145786667 | 145879292 |
| 59 | 2531377 | 2.7912053 | 2.971645 | 2.719586 | 4.743274 | 5.124374 | 4.30358 | 0.61749052 | 0.830476 | 0.4378769 | SP100 | chr2 | + | 230989257 | 231115964 |
| 60 | 3649811 | 2.7538157 | 2.753816 | 2.557624 | 4.4654356 | 4.465436 | 5.369619 | 0.49707286 | 0.4970729 | 1.004365 | NDE1 MYH11 | chr16 | + | 15651623 | 15725602 |
| 61 | 2743085 | 3.5189752 | 3.576003 | 2.14047 | 4.695039 | 5.064087 | 3.629734 | 0.59470365 | 0.7925065 | 0.2570947 | LARP2 | chr4 | + | 129215081 | 129363496 |
| 62 | 3538789 | 2.753609 | 2.751814 | 3.269128 | 4.3013298 | 4.398526 | 4.965744 | 0.43710475 | 0.4717085 | 0.7342142 | SLC38A6 | chr14 | + | 60517646 | 60588880 |
| 63 | 4008855 | 2.4515832 | 2.915424 | 3.146352 | 4.6079832 | 4.461829 | 4.681319 | 0.55565057 | 0.4956721 | 0.5883745 | SSX7 SSX9 SSX10 SSX6 SSX3 | chrX | - | 52689865 | 52700622 |
| 64 | 2394478 | 3.0352108 | 3.24743 | 2.447843 | 4.8604452 | 4.889646 | 3.657092 | 0.67648335 | 0.6920288 | 0.2627606 | CHD5 | chr1 | - | 6084494 | 6162755 |
| 65 | 2607055 | 2.479581 | 2.474251 | 2.678606 | 4.4907837 | 4.531962 | 4.843488 | 0.5070269 | 0.5236155 | 0.6676144 | PASK | chr2 | - | 241694411 | 241731162 |
| 66 | 3986230 | 1.6424152 | 3.314993 | 3.258358 | 2.6986094 | 5.176115 | 5.216128 | 0.12067416 | 0.8644917 | 0.8917384 | CXorf57 | chrX | + | 105741977 | 105809292 |
| 67 | 3107242 | 3.1206751 | 3.218324 | 2.159534 | 4.7596664 | 4.85342 | 3.529736 | 0.62542834 | 0.672795 | 0.237367 | TMEM67 | chr8 | + | 94836349 | 94900556 |
| 68 | 3374517 | 2.405991 | 2.460987 | 2.460987 | 4.7622573 | 4.603919 | 4.603919 | 0.6266921 | 0.5538905 | 0.5538905 | GLYATL2 | chr11 | - | 58358275 | 58368706 |
| 69 | 3301218 | 2.7627427 | 2.762743 | 2.215647 | 4.4235226 | 4.423523 | 4.810084 | 0.48103236 | 0.4810324 | 0.6504774 | PDLIM1 | chr10 | - | 96987333 | 97040767 |
| 70 | 2790486 | 2.6779806 | 2.713494 | 1.930721 | 5.1497287 | 5.107233 | 3.457851 | 0.8469763 | 0.8195013 | 0.224091 | DCHS2 | chr4 | - | 155375021 | 155632182 |
| 71 | 2396121 | 2.3085765 | 2.308577 | 1.838321 | 4.7740618 | 4.774062 | 4.748604 | 0.6324819 | 0.6324819 | 0.6200605 | DFFA | chr1 | - | 10443510 | 10455174 |
| 72 | 2857131 | 2.8571212 | 2.857121 | 2.978133 | 4.1224537 | 4.122454 | 4.36222 | 0.37978425 | 0.3797843 | 0.4584803 | DHX29 | chr5 | - | 54587849 | 54639138 |
| 73 | 2676182 | 2.4797585 | 2.48802 | 2.432348 | 4.5988309 | 4.740038 | 4.197833 | 0.55169422 | 0.6159352 | 0.402985 | STAB1 NT5DC2 | chr3 | - | 52533450 | 52544109 |
| 74 | 2800477 | 2.5419277 | 2.556754 | 2.238225 | 4.6330443 | 4.993802 | 3.734498 | 0.56662706 | 0.7503999 | 0.2794502 | SRD5A1 | chr5 | + | 6686561 | 6722612 |
| 75 | 3235516 | 2.647549 | 2.763852 | 1.879745 | 4.890948 | 4.891093 | 3.553364 | 0.69273002 | 0.6928081 | 0.2418928 | CAMK1D | chr10 | + | 12431641 | 12911466 |
| 76 | 2484970 | 2.8095684 | 2.840512 | 2.772546 | 4.1487072 | 4.188391 | 4.237921 | 0.38771306 | 0.4000049 | 0.4158805 | EHBP1 | chr2 | + | 62787607 | 63126945 |
| 77 | 3853108 | 3.2080623 | 3.064075 | 1.960494 | 4.4803765 | 4.504121 | 3.678088 | 0.50291664 | 0.5123427 | 0.2671901 | NOTCH3 | chr19 | - | 15131518 | 15172791 |
| 78 | 3165825 | 2.6604474 | 2.650323 | 2.601853 | 4.2191353 | 4.33286 | 4.306832 | 0.40978825 | 0.4480489 | 0.4389955 | TEK | chr9 | + | 27099492 | 27220140 |
| 79 | 2886174 | 2.2875653 | 2.266219 | 2.486764 | 4.2985232 | 4.392332 | 4.706237 | 0.43614335 | 0.4694256 | 0.5999187 | SLIT3 | chr5 | - | 168025894 | 168660537 |
| 80 | 3502259 | 2.4421599 | 2.47813 | 2.859145 | 3.8304642 | 4.098641 | 4.845112 | 0.30156504 | 0.3727293 | 0.6684588 | MCF2L | chr13 | + | 112682010 | 112798773 |
| 81 | 2891241 | 2.1115617 | 2.413024 | 2.501625 | 4.0507448 | 4.586015 | 4.660919 | 0.35892333 | 0.5462008 | 0.579086 | DUSP22 | chr6 | + | 237166 | 296339 |
| 82 | 3995392 | 2.2876188 | 2.309255 | 2.618431 | 4.3063268 | 4.389831 | 4.457079 | 0.43882161 | 0.4685066 | 0.4938333 | ZNF185 | chrX | + | 151837329 | 151892228 |
| 83 | 3527864 | 2.5420611 | 2.586287 | 2.776575 | 4.0198601 | 4.075105 | 4.510779 | 0.35028593 | 0.3658816 | 0.5150164 | FLJ10357 | chr14 | + | 20611070 | 20627409 |
| 84 | 2881239 | 2.5351407 | 2.582761 | 2.537905 | 3.9848868 | 4.022819 | 4.664307 | 0.34074883 | 0.3511045 | 0.5806188 | PDGFRB NIN | chr5 | - | 149473649 | 149496803 |
| 85 | 2880051 | 2.4466749 | 2.439136 | 2.23383 | 4.3868275 | 4.036126 | 4.610879 | 0.46740578 | 0.3548096 | 0.5569083 | PPP2R2B | chr5 | - | 145948379 | 146440878 |
| 86 | 2724853 | 2.1058198 | 2.632174 | 1.883837 | 3.8795652 | 4.863735 | 4.422963 | 0.31352495 | 0.6782172 | 0.4808217 | NSUN7 | chr4 | + | 40446829 | 40506371 |
| 87 | 3687475 | 2.3420955 | 2.333695 | 2.227138 | 4.1796482 | 4.344855 | 4.467327 | 0.39726467 | 0.4522822 | 0.4978091 | GDPD3 | chr16 | - | 30023662 | 30032300 |
| 88 | 3695648 | 2.5097872 | 2.459984 | 2.008408 | 4.7044304 | 4.42523 | 3.711655 | 0.59907445 | 0.4816756 | 0.2744216 | ZDHHC1 | chr16 | - | 65985843 | 66007761 |
| 89 | 4046876 | 2.3017802 | 2.30178 | 1.911378 | 4.2810622 | 4.281062 | 4.625708 | 0.43020829 | 0.4302083 | 0.5633922 | LOC727761 DTYMK | chr2_random | - | 102982 | 97500 |
| 90 | 3923537 | 2.4031979 | 2.403198 | 1.834163 | 4.3496875 | 4.349688 | 3.913031 | 0.4539988 | 0.4539988 | 0.3219384 | C21orf33 | chr21 | + | 44377949 | 44390007 |
| 91 | 3850660 | 2.5390282 | 2.281718 | 2.063976 | 3.4815342 | 4.303832 | 4.581673 | 0.22838437 | 0.4379637 | 0.5443517 | SPC24 | chr19 | - | 11117290 | 11127449 |
| 92 | 2369680 | 2.5465932 | 2.673792 | 2.020201 | 4.1644982 | 4.282095 | 3.615017 | 0.3925595 | 0.4305572 | 0.2540956 | TDRD5 | chr1 | + | 177827821 | 177926862 |
| 93 | 2559849 | 2.6917672 | 2.593286 | 2.125113 | 4.0902704 | 4.048925 | 3.684548 | 0.3702798 | 0.3584086 | 0.2685674 | SLC4A5 | chr2 | - | 74297140 | 74423155 |
| 94 | 3435980 | 2.5639757 | 2.460207 | 2.020853 | 3.9918327 | 4.052451 | 3.86393 | 0.34262267 | 0.3594065 | 0.3096676 | TCTN2 | chr12 | + | 122721744 | 122758859 |
| 95 | 4000839 | 2.4801478 | 2.522459 | 1.831146 | 3.9894913 | 4.104221 | 3.969817 | 0.3419899 | 0.3743709 | 0.3367176 | CTPS2 | chrX | - | 16516050 | 16640921 |
| 96 | 3327166 | 2.5029826 | 2.502983 | 1.809052 | 4.0567085 | 4.056708 | 3.926507 | 0.36061485 | 0.3606148 | 0.3253875 | C11orf74 | chr11 | + | 36572649 | 36637386 |
| 97 | 2406420 | 1.8200092 | 2.514155 | 1.997735 | 3.195466 | 4.112685 | 3.898894 | 0.18139155 | 0.3768745 | 0.3183579 | CLSPN | chr1 | - | 35958417 | 36003514 |
| 98 | 2440664 | 1.4831879 | 2.496798 | 2.475684 | 2.3078943 | 4.53304 | 4.037215 | 0.08675195 | 0.5240567 | 0.3551142 | B4GALT3 | chr1 | - | 159407871 | 159413938 |
| 99 | 3822322 | 0.9389246 | 2.38722 | 2.087168 | 1.7672152 | 4.894584 | 4.366155 | 0.05372224 | 0.6946922 | 0.4598964 | MRI1 | chr19 | + | 13736367 | 13746075 |
| 100 | 2798586 | 0.8846294 | 2.712624 | 2.529006 | 0.9767566 | 4.03299 | 4.227544 | 0.02413416 | 0.3539329 | 0.4125045 | PDCD6 LOC728613 AHRR | chr5 | + | 324806 | 488184 |
